# CircRNA, lncRNA, and mRNA profiles of umbilical cord blood exosomes from preterm newborns showing bronchopulmonary dysplasia

**DOI:** 10.1007/s00431-022-04544-2

**Published:** 2022-07-05

**Authors:** Yu Wang, Xuan Wang, Qiushi Xu, Jiao Yin, Huaiyan Wang, Lin Zhang

**Affiliations:** Department of Neonatology, Changzhou Maternal and Child Health Care Hospital, Changzhou, China

**Keywords:** Bronchopulmonary dysplasia, Exosome, circRNA, lncRNA, Umbilical cord blood

## Abstract

Bronchopulmonary dysplasia (BPD) represents a multifactorial chronic pulmonary pathology and a major factor causing premature illness and death. The therapeutic role of exosomes in BPD has been feverishly investigated. Meanwhile, the potential roles of exosomal circRNAs, lncRNAs, and mRNAs in umbilical cord blood (UCB) serum have not been studied. This study aimed to detect the expression profiles of circRNAs, lncRNAs, and mRNAs in UCB-derived exosomes of infants with BPD. Microarray analysis was performed to compare the RNA profiles of UCB-derived exosomes of a preterm newborn with (BPD group) and without (non-BPD, NBPD group) BPD. Then, circRNA/lncRNA–miRNA–mRNA co-expression networks were built to determine their association with BPD. In addition, cell counting kit-8 (CCK-8) assay was used to evaluate the proliferation of lipopolysaccharide (LPS)-induced human bronchial epithelial cells (BEAS-2B cells) and human umbilical vein endothelial cells (HUVECs). The levels of tumor necrosis factor (TNF)-α and interleukin (IL)-1β in LPS-induced BEAS-2B cells and HUVECs were assessed through Western blot analysis. Then, quantitative reverse transcription–polymerase chain reaction assay was used to evaluate the expression levels of four differentially expressed circRNAs (hsa_circ_0086913, hsa_circ_0049170, hsa_circ_0087059, and hsa_circ_0065188) and two lncRNAs (small nucleolar RNA host gene 20 (SNHG20) and LINC00582) detected in LPS-induced BEAS-2B cells or HUVECs. A total of 317 circRNAs, 104 lncRNAs, and 135 mRNAs showed significant differential expression in UCB-derived exosomes of preterm infants with BPD compared with those with NBPD. Gene Ontology (GO) enrichment and Kyoto Encyclopedia of Genes and Genomes (KEGG) pathway analyses were conducted to examine differentially expressed exosomal circRNAs, lncRNAs, and mRNAs. The results showed that the GO terms and KEGG pathways mostly involving differentially expressed exosomal RNAs were closely associated with endothelial or epithelial cell development. In vitro, CCK-8 and Western blot assays revealed that LPS remarkably inhibited the viability and promoted inflammatory responses (TNF-α and IL-1β) of BEAS-2B cells or HUVECs. The expression levels of circRNAs hsa_circ_0049170 and hsa_circ_0087059 were upregulated in LPS-induced BEAS-2B cells; the expression level of hsa_circ_0086913 was upregulated and that of hsa_circ_0065188 was downregulated in LPS-induced HUVECs. Moreover, the expression level of lncRNA SNHG20 was upregulated and that of LINC00582 was downregulated in LPS-induced BEAS-2B cells. Further, 455 circRNA/lncRNA–miRNA–mRNA interaction networks were predicted, including hsa_circ_0086913/hsa-miR-103a-3p/transmembrane 4 L six family member 1 (TM4SF1) and lncRNA-SNHG20/hsa-miR-6720-5p/spermine synthase (SMS) networks, which may take part in BPD.

*Conclusion*: This study provided a systematic perspective on UCB-derived exosomal circRNAs and lncRNAs and laid an important foundation for further investigating the potential biological functions of exosomal circRNAs and lncRNAs in BPD.

**Table:** 

**What is Known:** *• BPD represents a multifactorial chronic pulmonary pathology and a major factor causing premature illness and death.* *• The therapeutic role of exosomes in BPD has been feverishly investigated, and exosomal RNAs were ignored.*	
**What is New:** *• The profiles of UCB-derived exosomal circRNAs, lncRNAs, and mRNAs were performed.* *• Several differentially expressed circRNAs and lncRNAs were identified in LPS-induced BEAS-2B cells and HUVECs.*

**Supplementary Information:**

The online version contains supplementary material available at 10.1007/s00431-022-04544-2.

## Introduction

Bronchopulmonary dysplasia (BPD) represents a multifactorial chronic pulmonary pathology and a major factor causing premature illness and death, especially in premature infants with gestational age (GA) < 28 weeks [1]. The survival rate in extremely preterm infants has markedly increased with the progress in perinatal medicine; however, the morbidity of BPD has also increased. In the USA, the survival rate of infants with a GA of 22–28 weeks has increased from 70 to 79% in the past two decades. Meanwhile, the incidence of BPD has increased from 32 to 45% [2]. In Japan, the mortality rate of extremely preterm infants has decreased from 19.0% in 2003 to 8.0% in 2016, but the rate of BPD has increased from 41.40 to 52.0% among survivors [3]. Meanwhile, Chao Chen et al. found an increase in survival from 2010 (56.4%) to 2019 (67.1%) for infants born at a GA < 28 weeks, with BPD prevalence increasing from 55.7 to 79.9% among survivors in China [4]. From a pathophysiological point of view, aberrant reparative responses in the prenatal setting and recurrent postnatal injuries to the developing lungs jointly cause BPD [5]. In addition, the umbilical cord vein transports oxygenated blood with nutrition and other factors from the placenta to the developing fetus. Changes in certain nutrients and factors included in the umbilical cord blood (UCB) may have an important role in fetal programming, including lung development [6]. Assessing the substances contained in UCB may therefore help understand their influence on lung development and BPD.

Exosomes represent single-membrane organelles with 30–200-nm diameters secreted from cells [7] and can be obtained from UCB simultaneously [8–10]. Many researchers reported that exosomes played a crucial role in BPD [11–13], and may function through selected proteins, lipids, nucleic acids, and glycoconjugates [14, 15]. In addition, a recent study demonstrated that UCB-derived exosomes of infants with BPD impair angiogenesis, potentially through differentially expressed exosomal miRNAs [16]. However, the roles of UCB-derived exosomal circular RNAs (circRNAs) of infants with BPD remain poorly understood. As another class of noncoding RNAs, circRNAs regulate gene expression in eukaryotes and are involved in multiple pathologies, including cancer, cardiovascular diseases, and diabetes mellitus [17]. In addition, differentially expressed circRNAs have been detected in UCB-derived exosomes of patients with gestational diabetes mellitus (GDM) and preeclampsia, clearly suggesting pathological and developmental roles of exosomal circRNAs [18, 19]. Therefore, the present study applied microarrays to comparatively assess circRNA, lncRNA, and mRNA profiles of UCB-derived exosomes between preterm newborns with (BPD group) and without (NBPD group) BPD, aiming to provide a basis for more researches examining the role of exosomal circRNAs in BPD.

## Materials and methods

### Patients and samples

This descriptive study followed the recommendations of the ethics committee of the Changzhou Maternal and Child Health Care Hospital (approval number: 2021142) and was registered in the Chinese Clinical Trial Registry (approval number: ChiCTR2100049129). All participants and clinical data were collected from the Changzhou Maternal and Child Health Care Hospital from April to July 2021. BPD was defined as treatment with oxygen > 21% for at least 28 days as proposed by the National Institute of Child Health and Human Development [20]. The time point of assessment was 36-week postmenstrual age or discharge to home in infants with a GA < 32 weeks, or > 28 days but < 56 days postnatal age, or discharge to home in infants with a GA > 32 weeks, whichever came first [20]. The inclusion criteria were as follows: preterm infants without genetic or structural anomalies, delivered at less than 32 weeks of gestation, and showing BPD (BPD group) or not (NBPD group). The exclusion criteria were as follows: pregnant women with infectious diseases; neonates with severe heart and lung malformations; and patients with severe hypoxic–ischemic encephalopathy, abnormal development of the intracranial hemorrhagic brain, or chromosomal abnormalities. Finally, eight UCB specimens were obtained from the umbilical vein right after fetal delivery (four BPD and four NBPD preterm infants) for microarray screening, and another 20 specimens (ten BPD and ten NBPD preterm infants) were obtained for validation. After clipping the umbilical cord, 5 mL of umbilical venous blood was immediately extracted from the placental end using a syringe and placed in a vacuum blood collection tube containing coagulant and inert separation glue. Then, the blood was laid aside at room temperature for 1 h. After the blood was curdled and the light yellow transparent liquid was precipitated, the collected samples were centrifuged at 1000* g* at room temperature for 10 min. Finally, the supernatant, which was umbilical venous blood serum, was extracted into new Eppendorf tubes and stored at − 80 °C until further use.

### Isolation of exosomes from UCB serum

Exosomes were isolated following the protocol of ExoQuick exosome precipitation solution (cat. no. EXOTC50A-1 (5 mL), System Biosciences (SBI), CA, USA). First, 1 mL of UCB serum was centrifuged for 15 min at 3000* g* and 4 ℃ for the removal of cells and debris. The resulting serum was absorbed and added to 1.5-mL centrifuge tubes with 5 μL of thrombin (T4648-1KU, Sigma, MO, USA). After mixing, the samples were incubated for 15 min at 37 ℃. After centrifugation at 10,000* g* for 15 min at 4 ℃, the resulting supernatants were removed and the precipitated exosomes in the pellet were added with 250 μL of ExoQuick exosome precipitation solution. Then, specimens were mixed well and incubated for 30 min at 4 ℃. Exosomes were pelleted by 5-min centrifugation at 1500* g* at 4 ℃. The isolated exosomes were eluted in phosphate-buffered saline (PBS) and used immediately or stored at − 80 ℃ for later use.

### Nanoparticle tracking analysis

Exosome particle number was measured by nanoparticle tracking analysis (NTA) based on a previously published technique [21]. In brief, exosomes diluted in PBS were analyzed by nanoparticle tracking using the ZetaView (Particle Metrix, Germany) equipment. A 405-nm excitation laser was used in instruments precalibrated with a 100-nm PSL standard (Applied Microspheres, Netherlands). NTA was performed with the same camera settings and tracking parameters, appropriate for detecting extracellular vesicle (sensitivity, 85; shutter, 70 min; brightness, 20 min; size, 10; maximum size, 200). Video acquisition was carried out at 30 frames/s, and videos were assessed for size and concentration using ZetaView.

### Transmission electron microscopy

For transmission electron microscopy (TEM), exosomes pelleted by ultracentrifugation were resuspended in PBS. A drop thereof was placed on a copper mesh for 5 min. This was followed by 1-min staining with 1% phosphotungstic acid 44-hydrate and 20-min drying at room temperature. The preparations were examined under a transmission electron microscope (FEI, Tecnai G2 Spirit BioTwin; acceleration voltage, 80 kV).

### RNA purification from exosomes and microarrays

Total RNA extraction uses an miRNeasy Serum Kit (cat. no. 217184, QIAGEN, GmBH, Germany) as directed by the manufacturer. RNA integrity was examined on an Agilent Bioanalyzer 2100 (Agilent Technologies, CA, USA). Then, total RNA amplification and labeling used a Low Input Quick Amp Labeling Kit, One-Color (cat. no. 5190–2305, Agilent Technologies) according to the manufacturer’s protocol. Labeled circRNAs were obtained using an RNeasy Mini Kit (cat no. 74106, QIAGEN, GmBH).

The slides were hybridized using 1.65 μg of Cy3-labeled circRNA and a Gene Expression Hybridization Kit (cat. no. 5188–5242, Agilent Technologies) as directed by the manufacturer for 17 h. Staining dishes (cat. no. 121, Thermo Shandon, MA, USA) were used for washing with a Gene Expression Wash Buffer Kit (cat. no. 5188–5327, Agilent Technologies), according to the manufacturer’s protocol.

An Agilent Microarray Scanner (cat. no. G2565CA, Agilent Technologies) was used for scanning, with default settings. Data were extracted using Feature Extraction v10.7 (Agilent Technologies). Raw data were normalized using the Quantile algorithm and limma in R. Microarray analysis was carried out by Shanghai Biotechnology (China).

### Functional enrichment analyses

Ratios were calculated between four preterm infants with BPD and four with NBPD. Genes showing fold changes ≥ 2 and *P* < 0.05 (*t* test) were considered significantly differentially expressed. The chosen genes for exosomal circRNAs, lncRNAs, and mRNAs were analyzed using Gene Ontology (GO) enrichment and Kyoto Encyclopedia of Genes and Genomes (KEGG) with enrichment analysis software by Shanghai Biotechnology.

### CircRNA/lncRNA–miRNA–mRNA network building

The miRanda database was used for predicting circRNA/microRNA (miRNA) interactions based on miRNA response elements (MREs) on circRNAs, with miRanda v3.3a. MREs on circRNA/lncRNAs were retrieved, and miRNAs were selected according to the seed matching sequences. For lncRNAs and mRNAs paired with the identical miRNA, the Pearson correlation coefficient (PCC) was determined for identifying the inferred circRNA/lncRNA–miRNA–mRNA pairs. Then, circRNA/lncRNA–miRNA–mRNA pairs showing PCC ≥ 0.90 were included to construct a circRNA/lncRNA–miRNA–mRNA network.

### Cell culture and treatment

Human bronchial epithelial (BEAS-2B) cells and human umbilical vein endothelial cells (HUVECs) were provided by American Type Culture Collection (USA). These cells were routinely incubated in Dulbecco’s modified Eagle’s medium (Invitrogen, CA, USA) containing 10% fetal bovine serum (Invitrogen, Grand Island, NY, USA) and 1% penicillin–streptomycin (Sigma–Aldrich, MO, USA) at 37 °C with 5% CO_2_. The BEAS-2B cells were treated with lipopolysaccharide (LPS, 1 µg/mL) for 12 h, and HUVECs were treated with LPS (1 µg/mL) for 18 h.

### Cell counting kit-8 assay

For cell viability, the BEAS-2B cells and HUVECs were seeded in 96-well plates at a density of 1 × 10^4^ cells/well stimulated with LPS (1 µg/mL) for 12 h and 18 h, respectively. Then, a cell counting kit-8 (CCK-8) (Beyotime Biotechnology, China) was used to examine the cell viability, according to the manufacturer’s specification. The optical density was detected at 490 nm using a microplate reader (Tecan Infinite M200 Micro Plate Reader; LabX, Switzerland).

### Western blot analysis

Proteins extracted from BEAS-2B cells and HUVECs were measured using a bicinchoninic acid kit (Beyotime Biotechnology, China). Then, the proteins were resolved on sodium dodecyl sulfate–polyacrylamide gel electrophoresis (10%) and transferred to polyvinylidene fluoride (PVDF) membranes (Millipore, MA, USA). The PVDF membranes were incubated using 5% skimmed milk, and then with primary antibodies at 4 °C overnight. Blots were probed using the following antibodies: anti-IL-1β (1: 1, 000, ab234437; Abcam, Cambridge, UK), anti-TNF-α (1: 1, 000, ab183218; Abcam), and anti-glyceraldehyde-3-phosphate dehydrogenase (anti-GAPDH; 1: 2, 000, bs0755R; Bioss, China), with GAPDH being the endogenous control. Then, membranes were further incubated for 1 h using a secondary antibody (1:2, 000, b-0311P-HRP; Bioss).

### Quantitative real-time polymerase chain reaction

Based on relatively high abundance, FC ≥ 2.5, *P* < 0.01, and their host genes, we selected six differentially expressed RNAs to validate their expression in umbilical cord blood exosomes from additional ten BPD and ten NBPD preterm infants by quantitative real-time polymerase chain reaction (qRT-PCR) analysis, including three circRNAs (hsa_circ_0086913, hsa_circ_0007372 and hsa_circ_0065188) and three lncRNAs (membrane associated guanylate kinase, WW and PDZ domain-containing 2 (MAGI2) antisense RNA 3 (MAGI2-AS3), brain abundant membrane attached signal protein 1 antisense 1 RNA (BASP1-AS1), and solute carrier family 2 member 1 antisense RNA 1 (SLC2A1-AS1)).

After extracting total RNA from LPS-induced BEAS-2B cells and HUVECs, cDNA was prepared with RNA using an RNeasy plus micro kit, as the starting material of qPCR, carried out using a Step One System (Life Technologies Corp). Subsequently, four differentially expressed circRNAs (hsa_circ_0086913, hsa_circ_0049170, hsa_circ_0087059, and hsa_circ_0065188) and two lncRNAs (small nucleolar RNA host gene 20 (SNHG20) and LINC00582) selected based on the *P* value and fold change were evaluated by qRT-PCR analysis.

Primer Premier software 4.0 (Premier, Canada) was used to design sequences of all primers (see Table S1). GAPDH was normalized using the 2^−ΔΔCT^approach.

### Statistical analyses

SPSS 25.0 was used for data analysis. Quantitative data were expressed as mean ± standard deviation. Group pairs were compared using the Student *t* test. A *P* value < 0.05 indicated statistical significance.

## Results

### Description of preterm infants with BPD and NBPD

The demographic and clinical characteristics of preterm infants with BPD and NBPD are described in Tables 1 and 2. All the puerpera and newborns in this study belonged to the Han Chinese nationality and the yellow race. Specifically, birth weight, GA, gender, Apgar score, intraventricular hemorrhage, patent ductus arteriosus, respiratory distress syndrome, necrotizing enterocolitis, early-onset neonatal sepsis, late-onset neonatal sepsis, retinopathy of prematurity, surfactant treatment, mother with preeclampsia, antenatal steroids, premature rupture of membranes, and chorioamnionitis were similar in both groups. In addition, the continuous positive airway pressure, oxygen inhalation, and hospitalization days of preterm infants were longer in the BPD group than in the NBPD group.

**Table 1:** Clinical characteristics of the BPD and non-BPD infants in the microarray analysis

Group	BPD (*n* = 4)					NBPD (*n* = 4)					*P*
	1#	2#	3#	4#	(mean ± SEM)/*n*%	1#	2#	3#	4#	(mean ± SEM)/*n*%	
Infants’ characteristics											
Sex gender	Female	Male	Male	Female	2(50%)	Male	Female	Male	Female	2(50%)	1.00
BW (g)	1580	1350	1340	1250	1380.00 ± 140.71	1450	1580	1580	1830	1610.00 ± 158.95	0.07
GA (week)	31 + 6 (31.86)	29 + 6 (29.86)	28 + 2 (28.29)	27 + 6 (27.86)	29.47 ± 1.81	29 + 1 (29.14)	30 + 4 (30.57)	30 + 6 (30.86)	30 + 6 (30.86)	30.36 ± 0.41	0.41
Apgar 1 min	8	7	6	8	7.25 ± 0.96	6	5	8	8	7.00 ± 1.41	0.78
Apgar 5 min	8	8	8	8	8.00 ± 0.00	7	8	8	8	7.75 ± 0.50	0.39
IVH	Yes	Yes	Yes	Yes	4(100%)	Yes	Yes	Yes	Yes	4(100%)	0.13
RDS	Yes	No	Yes	No	2(50%)	No	No	Yes	Yes	2(50%)	1.00
PDA	Yes	Yes	Yes	No	3(75%)	Yes	Yes	Yes	Yes	4(100%)	0.29
NEC	No	No	No	No	0(0%)	No	No	No	No	0(0%)	0.13
ROP	No	Zone III, stage 1, plus ( −)	Zone III, stage 2, plus ( −)	No	2(50%)	No	Zone III, stage 2, plus ( −)	No	No	1(25%)	0.47
EOS	No	No	Yes	No	1(25%)	No	No	No	No	0(0%)	0.29
LOS	Yes	No	No	No	1(25%)	Yes	No	No	No	1(25%)	1.00
PS treatment	Yes	Yes	Yes	Yes	4(100%)	Yes	No	Yes	Yes	3(75%)	0.29
IMV (day)	15	11	7	0	8.25 ± 6.40	4	0	0	7	2.75 ± 3.40	0.18
CPAP (day)	16	10	14	10	12.50 ± 3.00	6	3	3	4	4.00 ± 1.41	0.01
Days with oxygen (day)	57	61	76	32	56.50 ± 18.27	21	11	7	11	12.50 ± 5.97	0.00
Hospitalization days (day)	59	69	77	50	63.75 ± 11.76	56	37	33	40	41.50 ± 10.08	0.03
Maternal characteristics											
Preeclampsia	No	No	No	No	0(0%)	No	No	No	Yes	1(25%)	0.29
Antenatal steroids	Yes	Yes	Yes	Yes	4(100%)	No	Yes	Yes	Yes	3(75%)	0.29
PROM	Yes	Yes	No	No	2(50%)	No	No	No	No	0(0%)	2.67
Chorioamnionitis	Yes	No	Yes	No	2(50%)	No	No	Yes	No	1(25%)	0.47

*BW* birthweight, *GA* gestational age, *IVH* intraventricular hemorrhage, *RDS* respiratory distress syndrome, *PDA* patent ductus arteriosus, *NEC* necrotizing enterocolitis, *ROP* retinopathy of prematurity, *EOS* early-onset neonatal sepsis, *LOS* late-onset neonatal sepsis, *PS* pulmonary surfactant, *CPAP* continuous positive airway pressure, *IMV* invasive mechanical ventilation, *PROM* premature rupture of membranes

**Table 2:** Clinical characteristics of the BPD and non-BPD infants in the qRT-PCR quantification of circRNAs and lncRNAs

	BPD(*n* = 10)		NBPD(*n* = 10)		*P* value
	Mean ± SEM	*n*(%)	Mean ± SEM	*n*(%)	
Infants’ characteristics					
Male gender		7(70%)		7(70%)	1.000
BW (g)	1199.00 ± 183.09		1222.00 ± 156.76		0.766
GA (week)	28.53 ± 0.74		29.03 ± 1.97		0.468
Apgar 1 min	6.30 ± 1.49		6.20 ± 2.04		0.902
Apgar 5 min	7.30 ± 0.94		7.50 ± 0.71		0.600
IVH		8(80%)		10(100%)	0.136
RDS		8(80%)		8(80%)	1.000
PDA		7(70%)		7(70%)	1.000
NEC		1(10%)		0(0%)	0.305
ROP		5(50%)		4(40%)	0.653
EOS		1(10%)		2(20%)	0.531
LOS		5(50%)		4(40%)	0.653
PS treatment		8(80%)		9(90%)	0.531
IMV (day)	10.60 ± 9.57		3.50 ± 5.87		0.035
CPAP (day)	12.20 ± 5.55		5.00 ± 3.20		0.003
Days with oxygen (day)	44.90 ± 13.74		14.8 ± 10.39		< 0.001
Hospitalization days (day)	57.60 ± 9.30		40.2 ± 18.61		0.016
Maternal characteristics					
Preeclampsia		3(30%)		4(40%)	0.639
Antenatal steroids		8(80%)		8(80%)	1.000
PROM		3(30%)		2(20%)	0.606
Chorioamnionitis		4(40%)		4(40%)	1.000

*BW* birthweight, *GA* gestational age, *IVH* intraventricular hemorrhage, *RDS* respiratory distress syndrome, *PDA* patent ductus arteriosus, *NEC* necrotizing enterocolitis, *ROP* retinopathy of prematurity, *EOS* early-onset neonatal sepsis, *LOS* late-onset neonatal sepsis, *PS* pulmonary surfactant, *CPAP* continuous positive airway pressure, *IMV* invasive mechanical ventilation, *PROM* premature rupture of membranes

### Detection of exosomes

NTA and TEM analyses were performed for identifying the purified exosomes. The cup-shaped morphology and clear, intact membrane were identified by TEM (Fig. S1A), and particles between 30 and 120 nm were detected by NTA (Fig. S1B). These findings indicated that the serum-derived particles isolated from the study subjects were exosomes. No differences were observed in the size distribution and morphology between exosomes isolated from the BPD and NBPD groups.

### Exosomal circRNA, lncRNA, and mRNA profiles by microarray analysis of UCB in the BPD and NBPD groups

**Fig. 1:**
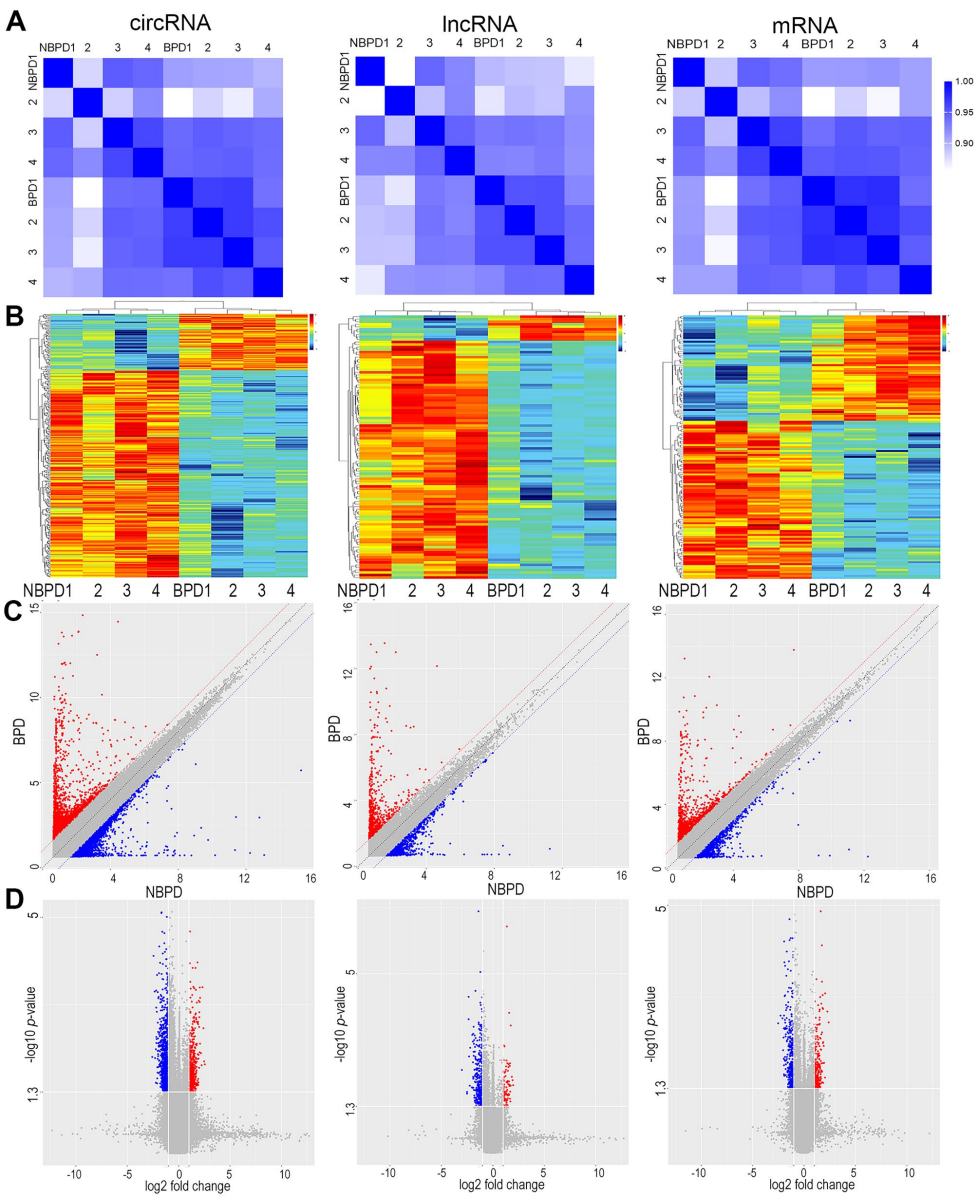
circRNAs, lncRNAs, and mRNAs with differential expression in umbilical cord blood exosomes between BPD and NBPD newborns. (**A**) Correlations among the eight specimens according to the expression of significantly regulated circRNAs, lncRNAs, and mRNAs. (**B**) Clustering heatmap of differentially expressed circRNAs, lncRNAs, and mRNAs. (**C**, **D**) Scatter and volcano plots depicting RNAs with differential expression between the BPD and NBPD groups. Red and blue represent upregulated and downregulated RNAs, respectively

A total of 105,509 circRNAs, 32,953 lncRNAs, and 34,549 mRNAs were identified as indicated in Fig. 1(A). Of these, 317 circRNAs, 104 lncRNAs, and 135 mRNAs showed significant differential expression based on the aforementioned criteria in UCB-derived exosomes of preterm infants in the BPD group compared with those in the NBPD group. Among them, 68 and 249 circRNAs, 94 and 10 lncRNAs, and 81 and 54 mRNAs were upregulated and downregulated, respectively, as presented in Fig. 1 and Table 3. The heatmap analysis disclosed low internal variation, suggesting that these gene alterations may be meaningful for BPD pathogenesis (Fig. 1(B)). Further, differentially expressed exosomal circRNAs, lncRNAs, and mRNAs were used to generate scatter (Fig. 1(C)) and volcano (Fig. 1(D)) plots.

**Table 3:** Differentially expressed circRNA, lncRNA, and mRNA in UCB-derived exosomes from the BPD and NBPD groups

Gene type	TargetID	*P* values	Fold change	Regulation	Host gene/gene symbol
circRNA	hsa_circ_0049170	0.007549	4.491964	Up	OLFM2
circRNA	hsa_circ_0087059	0.015400	3.942138	Up	GRHPR
circRNA	hsa_circ_0118639	0.009878	3.811908	Up	TRAK2
circRNA	hsa_circ_0132613	0.026462	3.729238	Up	RNGTT
circRNA	hsa_circ_0086913	0.007987	3.598227	Up	TLN1
circRNA	hsa_circ_0009420	0.033789	3.533802	Up	LOC388588
circRNA	hsa_circ_0061761	0.032548	3.338253	Up	WRB
circRNA	hsa_circ_0002540	0.019826	3.277644	Up	TCONS_l2_00004567
circRNA	hsa_circ_0013996	0.036328	3.202700	Up	PLEKHO1
circRNA	hsa_circ_0073391	0.015983	3.101576	Up	MCTP1
circRNA	hsa_circ_0092030	0.027085	3.039648	Up	FLNA
circRNA	hsa_circ_0036403	0.031816	2.977274	Up	ETFA
circRNA	hsa_circ_0138092	0.032494	2.957627	Up	ZER1
circRNA	hsa_circ_0018712	0.011858	2.893296	Up	PSAP
circRNA	hsa_circ_0036407	0.040910	2.857984	Up	ETFA
circRNA	hsa_circ_0075931	0.001353	2.810139	Up	–
circRNA	hsa_circ_0018723	0.039450	2.625034	Up	PSAP
circRNA	hsa_circ_0063129	0.001627	2.607178	Up	MYH9
circRNA	hsa_circ_0037578	0.010280	2.547076	Up	TCEB2
circRNA	hsa_circ_0036509	0.040011	2.539615	Up	ZFAND6
circRNA	hsa_circ_0007372	0.006241	4.642343	Down	IFT46
circRNA	hsa_circ_0028145	0.025837	4.246691	Down	TRPV4
circRNA	hsa_circ_0078460	0.010902	3.802345	Down	TULP4
circRNA	hsa_circ_0065188	0.000092	3.494472	Down	PTPN23
circRNA	hsa_circ_0037782	0.011926	3.353644	Down	METTL22
circRNA	hsa_circ_0034846	0.018938	3.155511	Down	CDAN1
circRNA	hsa_circ_0045828	0.007738	3.142472	Down	MXRA7
circRNA	hsa_circ_0084333	0.004997	3.140422	Down	PRKDC
circRNA	hsa_circ_0086018	0.008154	3.138347	Down	BOP1
circRNA	hsa_circ_0081368	0.025939	3.122619	Down	ZNF498
circRNA	hsa_circ_0028505	0.011100	3.087724	Down	SLC24A6
circRNA	hsa_circ_0038205	0.026746	3.065186	Down	–
circRNA	hsa_circ_0034360	0.018680	3.001853	Down	AQR
circRNA	hsa_circ_0028200	0.000758	2.993999	Down	ANAPC7
circRNA	hsa_circ_0011901	0.010229	2.977045	Down	RIMS3
circRNA	hsa_circ_0026805	0.000338	2.947501	Down	RPS26
circRNA	hsa_circ_0014196	0.000098	2.906941	Down	RORC
circRNA	hsa_circ_0111747	0.005713	2.870392	Down	PIK3C2B
circRNA	hsa_circ_0023216	0.002814	2.842620	Down	LRP5
circRNA	hsa_circ_0077639	0.006088	2.832914	Down	SLC16A10
lncRNA	ENST00000613892	3.605620	3.605620	Up	MAGI2-AS3
lncRNA	ENST00000566583	3.177118	3.177118	Up	SNHG20
lncRNA	ENST00000456953	2.657584	2.657584	Up	SNHG17
lncRNA	ENST00000434411	2.584309	2.584309	Up	SNHG20
lncRNA	ENST00000430373	2.432956	2.432956	Up	INKA2-AS1
lncRNA	ENST00000458314	2.284379	2.284379	Up	ITGA6-AS1
lncRNA	ENST00000505718	2.275216	2.275216	Up	ARHGAP22-IT1
lncRNA	ENST00000616527	2.097051	2.097051	Up	MALAT1
lncRNA	ENST00000663422	2.029238	2.029238	Up	MAGI2-AS3
lncRNA	ENST00000662112	2.013230	2.013230	Up	AC068733.3
lncRNA	ENST00000502301	0.269295	3.713394	Down	LINC00461
lncRNA	ENST00000553812	0.284493	3.515019	Down	AC008056.2
lncRNA	ENST00000671622	0.295918	3.379320	Down	LINC01094
lncRNA	ENST00000661332	0.310585	3.219735	Down	BASP1-AS1
lncRNA	ENST00000442305	0.325902	3.068406	Down	AL139246.4
lncRNA	LINC01467:4	0.344071	2.906373	Down	LINC01467
lncRNA	ENST00000444346	0.345258	2.896384	Down	LINC01983
lncRNA	ENST00000555433	0.353812	2.826360	Down	AL356022.1
lncRNA	ENST00000431759	0.358385	2.790299	Down	SLC2A1-AS1
lncRNA	ENST00000448058	0.362807	2.756284	Down	LINC00582
lncRNA	TEX41:26	0.368298	2.715191	Down	TEX41
lncRNA	ENST00000668542	0.379396	2.635771	Down	LINC01322
lncRNA	ENST00000447119	0.386033	2.590454	Down	UNC5B-AS1
lncRNA	ENST00000659430	0.386040	2.590404	Down	LINC01322
lncRNA	ENST00000553321	0.387800	2.578649	Down	AC068831.2
lncRNA	ENST00000663040	0.395291	2.529780	Down	AP001981.2
lncRNA	ENST00000577850	0.395566	2.528020	Down	AC002094.2
lncRNA	ENST00000657104	0.396799	2.520169	Down	LINC00308
lncRNA	LINC01149:1	0.401669	2.489613	Down	LINC01149
lncRNA	ENST00000655586	0.402178	2.486460	Down	LINC01322
mRNA	NM_005252	0.002311	5.254707	Up	FOS
mRNA	NM_003662	0.004022	3.217883	Up	PIR
mRNA	NM_004345	0.039567	3.111624	Up	CAMP
mRNA	NM_004417	0.004903	3.030365	Up	DUSP1
mRNA	NM_001267608	0.023351	2.939260	Up	FAM189B
mRNA	NM_000117	0.016202	2.882916	Up	EMD
mRNA	NM_005332	0.006985	2.838787	Up	HBZ
mRNA	NM_001145033	0.038541	2.699928	Up	C11orf96
mRNA	NM_022167	0.001038	2.583704	Up	XYLT2
mRNA	NM_003720	0.015725	2.569150	Up	PSMG1
mRNA	NM_014220	0.025066	2.527983	Up	TM4SF1
mRNA	NM_021213	0.011023	2.520859	Up	PCTP
mRNA	NM_003546	0.014696	2.504952	Up	HIST1H4L
mRNA	NM_030572	0.012157	2.459270	Up	SPX
mRNA	NM_001172415	0.045366	2.429838	Up	BAG1
mRNA	NM_001004318	0.009948	2.372999	Up	ACP7
mRNA	NM_032470	0.003170	2.330444	Up	TNXB
mRNA	NM_005332	0.016376	2.329259	Up	HBZ
mRNA	NM_000476	0.003512	2.320396	Up	AK1
mRNA	NM_001178056	0.049708	2.310605	Up	PARP8
mRNA	NM_198696	0.008439	4.096204	Down	KRTAP10-3
mRNA	NM_020994	0.000142	3.775682	Down	CTAG2
mRNA	NM_001164405	0.016215	3.428569	Down	BHLHA9
mRNA	ENST00000417284	0.001892	3.242241	Down	RGPD4-AS1
mRNA	ENST00000519609	0.014836	2.988594	Down	RP11-32D16.1
mRNA	ENST00000527997	0.006348	2.774404	Down	RP13-631K18.5
mRNA	NM_032862	0.000462	2.750719	Down	TIGD5
mRNA	NM_022822	0.003375	2.718796	Down	KLC2
mRNA	NM_001195520	0.046637	2.699180	Down	LRCOL1
mRNA	NM_024902	0.000019	2.666748	Down	DNAJC22
mRNA	NM_001277372	0.007239	2.640840	Down	KIAA2012
mRNA	NM_024522	0.016315	2.617907	Down	NKAIN1
mRNA	NM_001008409	0.005572	2.609773	Down	TTLL9
mRNA	ENST00000441860	0.004645	2.607226	Down	RPL23AP76
mRNA	ENST00000566382	0.003392	2.569043	Down	LARP4P
mRNA	NM_022752	0.002576	2.568464	Down	ZNF574
mRNA	ENST00000564204	0.005544	2.567630	Down	KIFC3
mRNA	NM_001018078	0.034002	2.554457	Down	FPGS
mRNA	NM_032512	0.002354	2.534126	Down	PDZD4
mRNA	ENST00000422723	0.005925	2.531463	Down	LINC01122

**Fig. 2:**
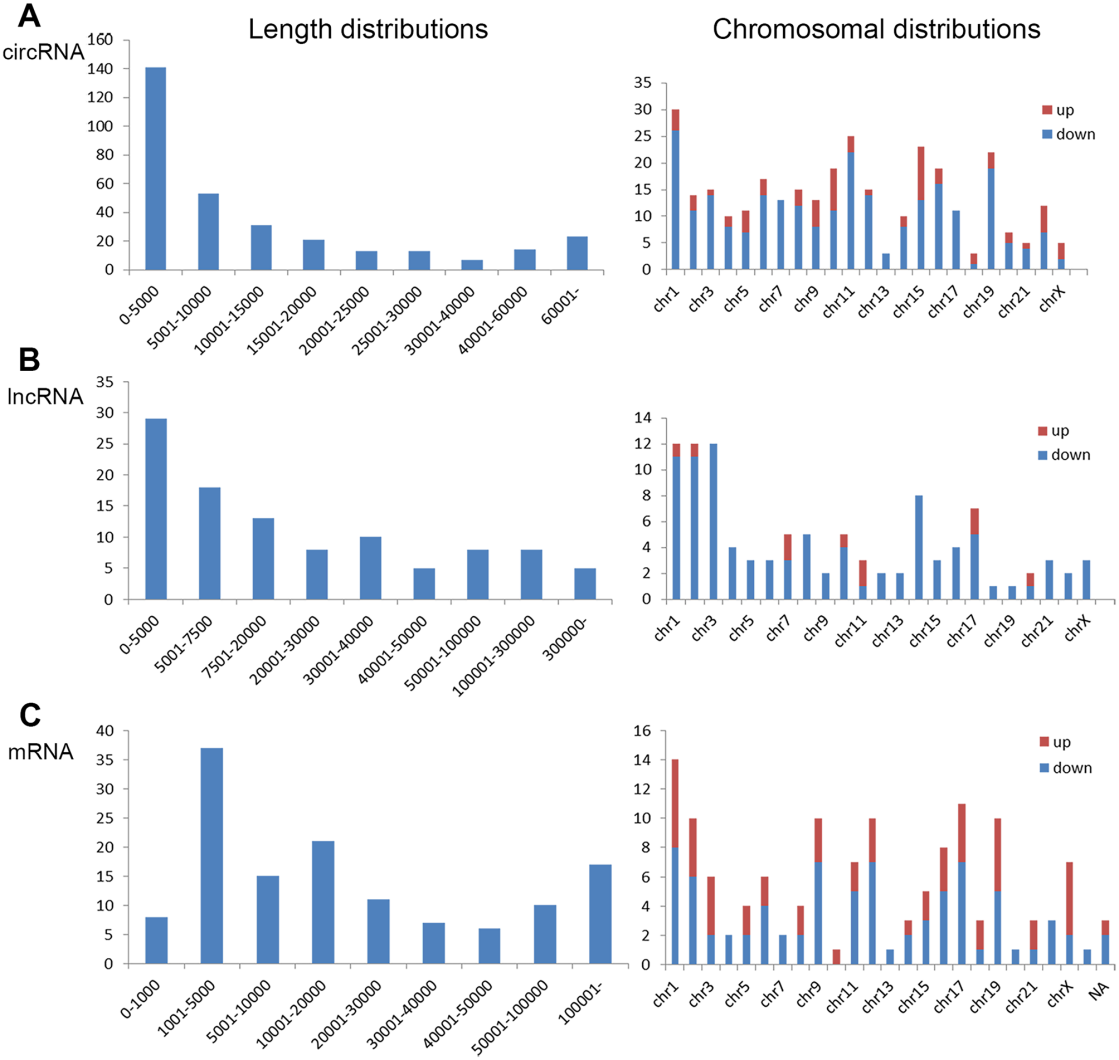
General characteristics of differentially expressed circRNAs, lncRNAs, and mRNAs in umbilical cord blood exosomes between the BPD and NBPD groups. Length distributions (left) and chromosomal distributions (right) of differentially expressed circRNAs (**A**), lncRNAs (**B**), and mRNAs (**C**). The *X*- and *Y*-axes represent gene length or chromosome and gene number, respectively

Next, differentially expressed circRNAs, lncRNAs, and mRNAs in UCB-derived exosomes were assessed for general features by preliminarily analyzing microarray data. Figure 2 shows that the parent genes of these circRNAs, lncRNAs, and mRNAs were broadly distributed in virtually all human chromosomes, also depicting their length distributions.

### Validation of differentially expressed circRNAs and lncRNAs by qRT-PCR

**Fig. 3:**
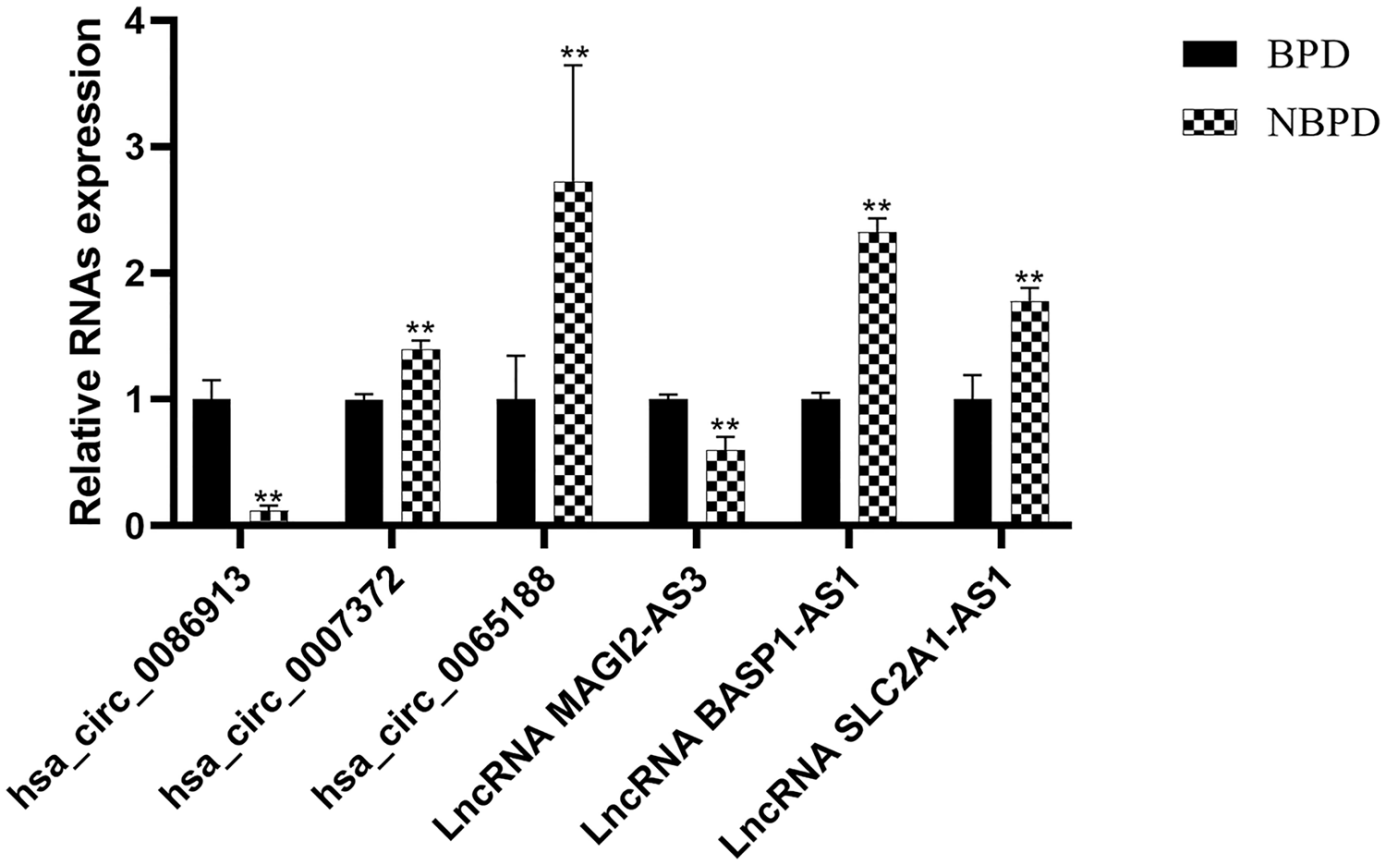
Validation of differentially expressed circRNAs and lncRNAs by qRT-PCR. CircRNAs hsa_circ_0086913, hsa_circ_0007372, and hsa_circ_0065188, and LncRNAs MAGI2-AS3, BASP1-AS,1 and SLC2A1-AS1 assessed by qRT-PCR in umbilical cord blood exosomes between BPD and NBPD newborns. (*n* = 10 per group, **P < 0.01)

In parallel with the microarray data, qRT-PCR results showed that the expressions of circRNA hsa_circ_0086913 and lncRNA MAGI2-AS3 were upregulated, and the expressions of circRNAs hsa_circ_0007372 and hsa_circ_0065188, and lncRNAs BASP1-AS1 and SLC2A1-AS1, were downregulated in the BPD group (Fig. 3).

### GO and KEGG analyses of exosomal circRNAs, lncRNAs, and mRNAs

**Fig. 4:**
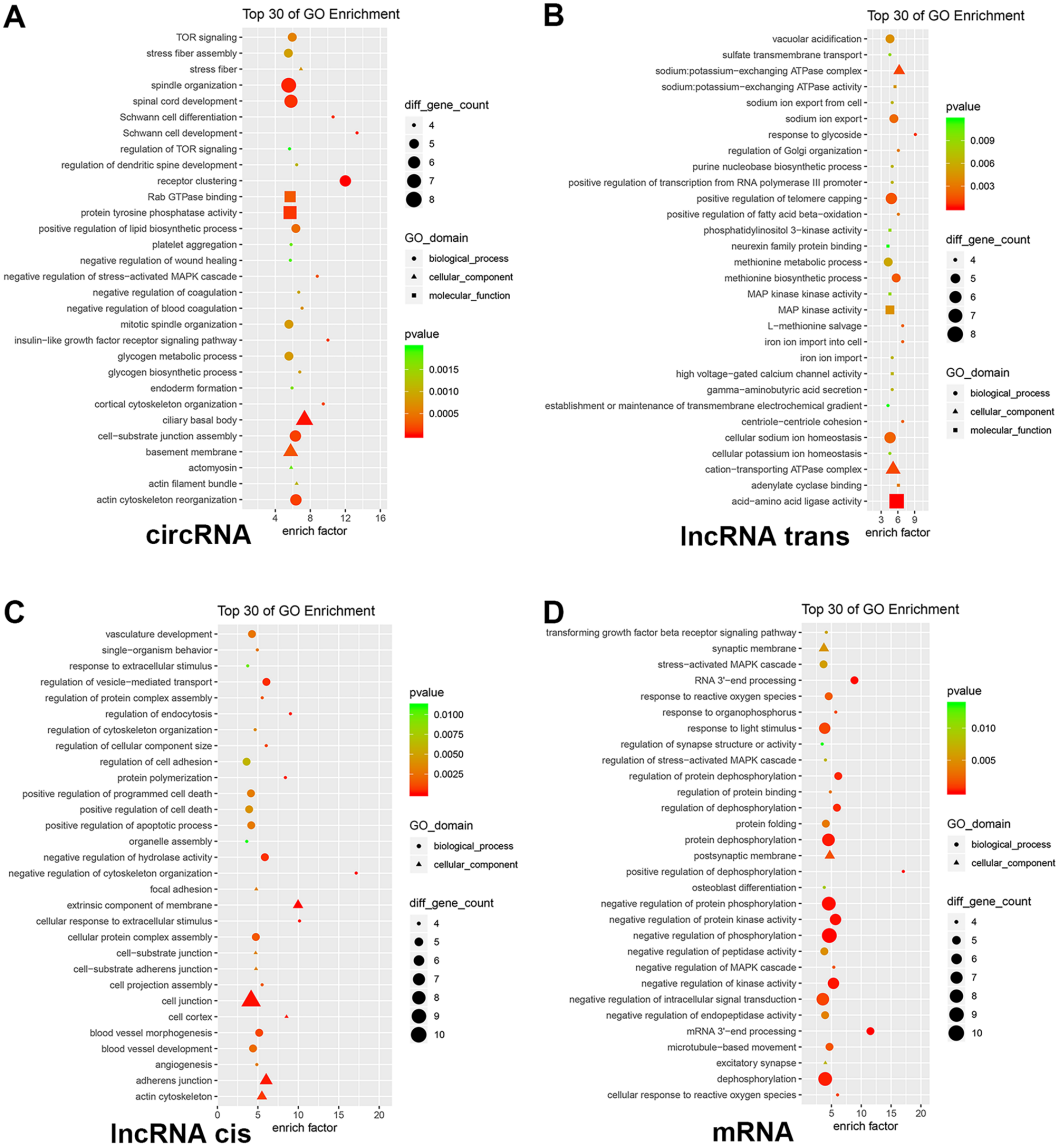
GO analyses circRNAs, lncRNAs and miRNAs with differential expression. Scatter plots of top 30 enriched GO terms involving circRNAs (**A**), lncRNAs-trans (**B**), lncRNAs-cis (**C**), and miRNAs (**D**) with differential expression. Ordinates represent GO terms, and abscissas are richness factors (richness factor = number of differentially expressed RNAs annotated to various terms/number of RNAs annotated to various terms). Dots reflect and are proportional to the amounts of RNAs with significant differential expression. *Q* values (0–1) are corrected *P* values. Points of different colors reflect distinct *Q* values

**Fig. 5:**
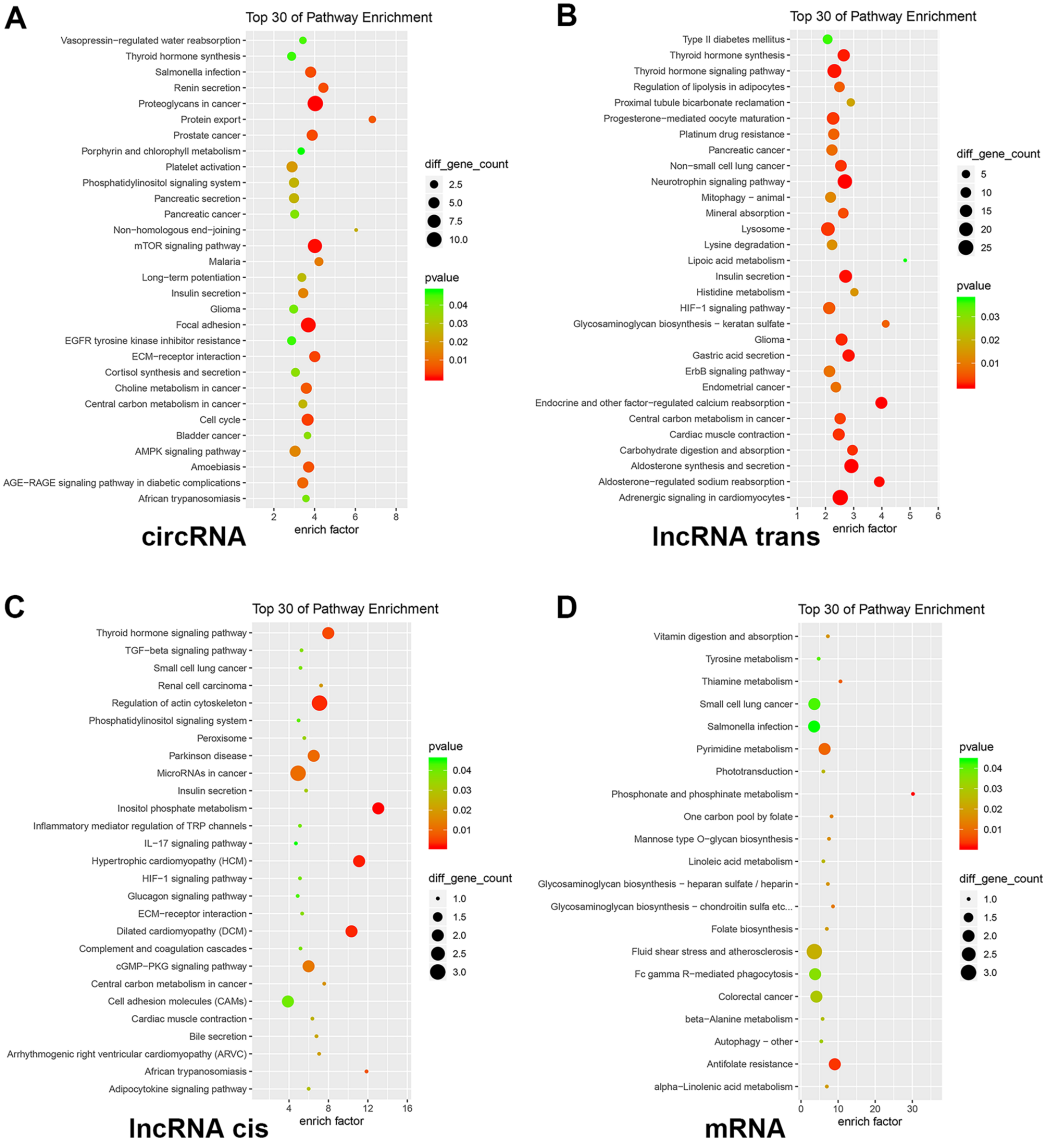
KEGG analyses circRNAs, lncRNAs, and miRNAs with differential expression. Scatter plots of top 30 enriched KEGG pathways involving circRNAs (**A**), lncRNAs-trans (**B**), lncRNAs-cis (**C**), and miRNAs (**D**) with differential expression. Ordinates represent pathway types, and abscissas are richness factors (richness factor = number of differentially expressed RNAs annotated to various terms/number of RNAs annotated to various terms). Dots reflect and are proportional to the amounts of RNAs with significant differential expression. *Q* values (0–1) are corrected *P* values. Points of different colors reflect distinct *Q* values

**Fig. 6:**
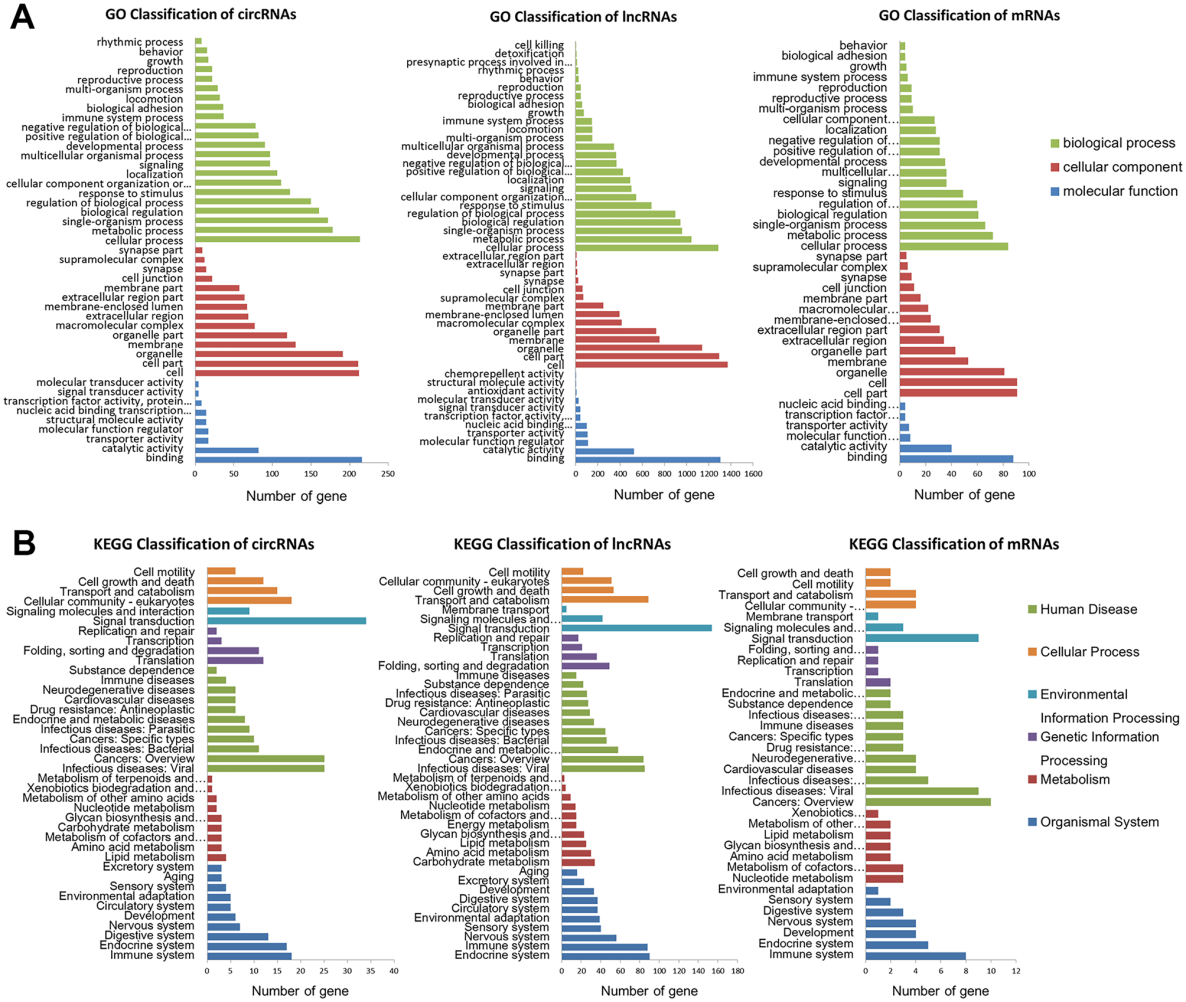
Classification of enriched GO functions and KEGG pathways. **A** Classification of enriched GO functions. The amounts of differentially expressed genes enriched in biological processes, cellular components and molecular function are shown. Abscissas and ordinates indicate GO terms and the amounts (and proportions) of genes enriched in various GO terms, respectively. **B** Classification of differentially KEGG pathways. The numbers of differentially expressed genes enriched in cellular processes, environmental information processing, genetic information processing, human diseases, metabolism, and organismal systems are shown. Abscissas and ordinates indicate KEGG pathways and numbers (and proportions) of genes enriched in various KEGG pathways, respectively

GO and KEGG analyses were executed for investigating the potential functions of differentially expressed genes (Figs. 4–6). The GO analysis revealed enrichment of 1165 circRNAs, 2475 lncRNAs, and 395 mRNAs in different physiological functions, such as molecular functions, cellular components, and biological processes (Fig. 4). Meanwhile, exosomal circRNAs, lncRNAs, and mRNAs were involved in 221, 365, and 135 KEGG pathways, respectively. Figure 5 shows the top 30 enriched KEGG terms of differentially expressed exosomal circRNAs, lncRNAs, and mRNAs between the BPD and NBPD groups. GO function and KEGG classifications of differentially expressed RNAs are shown in Fig. 6. The classification of KEGG included Organismal System, Metabolism, Human Disease, Genetic Information Processing, Environmental Information Processing, and Cellular Process, as shown in Fig. 6B.

### Prediction of exosomal circRNA/lncRNA–miRNA–mRNA interactions

**Fig. 7:**
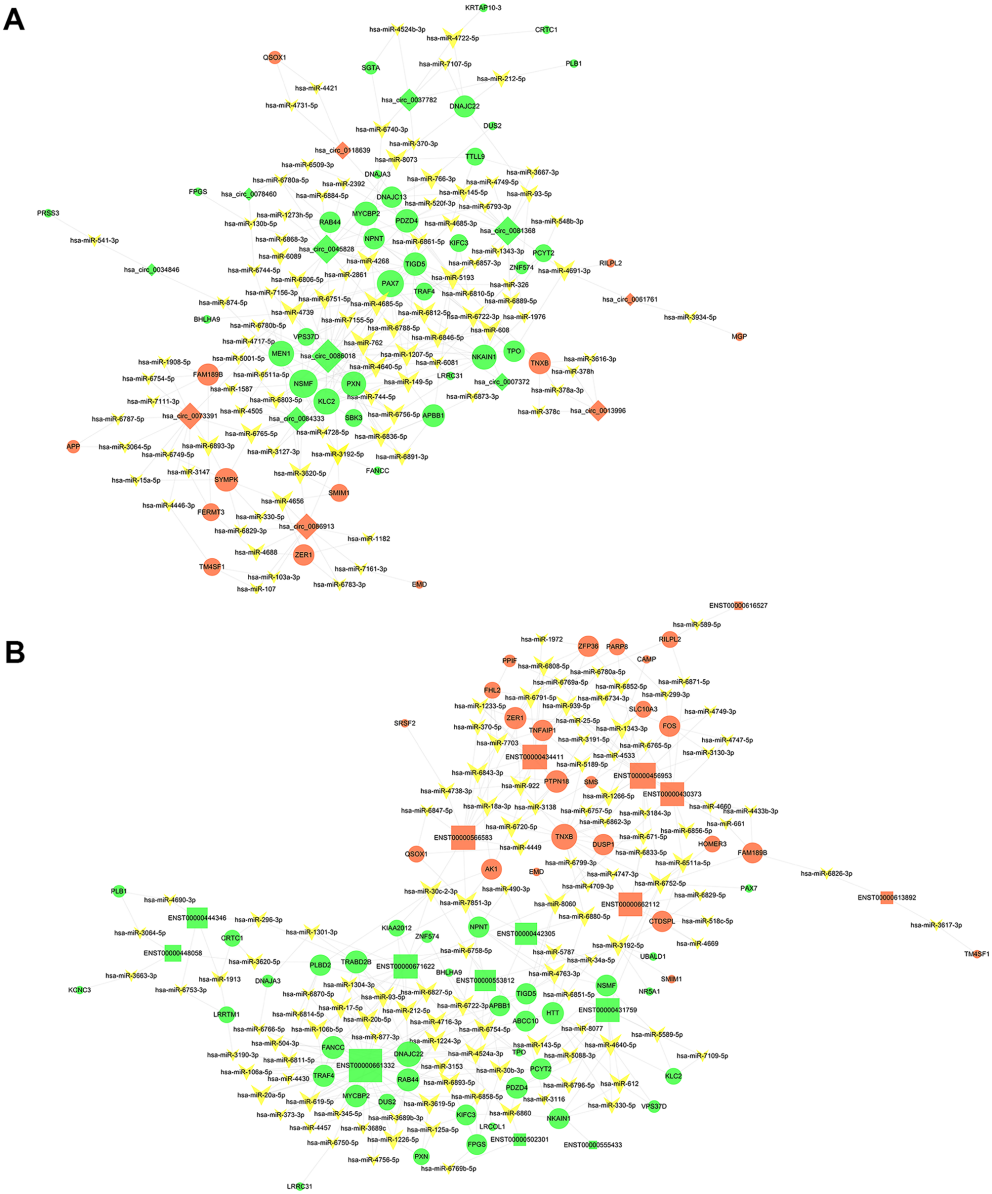
circRNA/lncRNA–miRNA–mRNA regulatory network in BPD. **A** Interaction network of circRNA–miRNA–mRNA predicted with PPC > 0.90. **B** Interaction network of lncRNA–miRNA–mRNA predicted with PPC > 0.90. Rhombuses, squares, circles, and triangles denote circRNAs, lncRNAs, mRNAs, and miRNAs, respectively. Red and green nodes reflect up- and downregulation, respectively; yellow nodes are undefined cases. The size of each node is proportional to the degree of involvement

circRNAs and lncRNAs have been shown to possess multiple binding sites for miRNAs, which they sponge, thus relieving the inhibitory effects of miRNAs on their target mRNAs. This effect is known as the competitive endogenous RNA (ceRNA) mechanism [22–24]. Therefore, miRanda was used to predict the potential ceRNAs of the first 10 upregulated and downregulated circRNAs and lncRNAs based on MREs, respectively. A total of 13 circRNAs, 97 miRNAs, and 45 mRNAs were retrieved (Fig. 7A). A total of 187 circRNA/lncRNA–miRNA–mRNA regulations with PPC > 0.90 were predicted as shown in Table S1. Moreover, 268 regulations existed among 164 transcripts that included 16 lncRNAs, 119 miRNAs, and 59 mRNAs, as shown in Fig. 7B and Table S2.

### Expression levels of differentially expressed circRNAs and lncRNAs in LPS-induced BEAS-2B cells and HUVECs

**Fig. 8:**
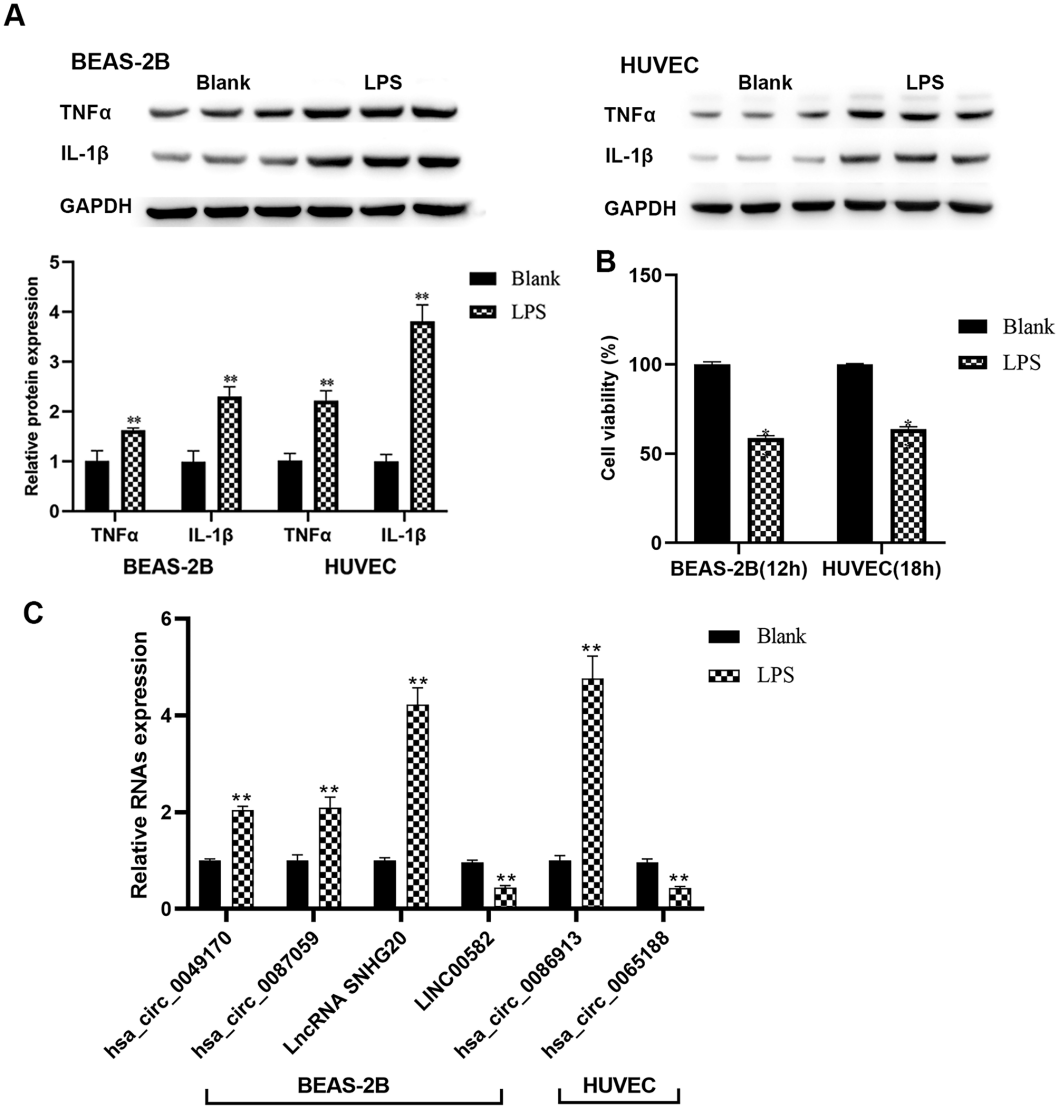
Expression levels of differentially expressed circRNAs and lncRNAs in LPS-induced BEAS-2B cells and HUVECs. **A** Expression levels of TNF-α and IL-1β evaluated by Western blot in LPS-induced BEAS-2B cells and HUVECs. **B** Cell proliferation of LPS-induced BEAS-2B cells and HUVECs evaluated by CCK-8 assay. **C** CircRNAs hsa_circ_0049170, hsa_circ_0087059, and lncRNAs SNHG20, LINC00582 assessed by qRT-PCR in LPS-induced BEAS-2B cells. And circRNAs hsa_circ_0086913 and hsa_circ_0065188 assessed by qRT-PCR in LPS-induced HUVECs. (*n* = 3 biological independent samples per group in qRT-PCR and Western blot, **P < 0.01)

LPS-induced injury in BEAS-2B cells and HUVECs is related to increased inflammation. As observed, TNF-α and IL-1β were higher in BEAS-2B cells and HUVECs after the stimulation of LPS, as analyzed by Western blot (Fig. 8A). Moreover, LPS also significantly inhibited the cell viability of BEAS-2B cells and HUVECs (Fig. 8B).

According to the high fold change, expression level, and host gene/gene symbol, four differentially expressed circRNAs (hsa_circ_0049170, hsa_circ_0087059, hsa_circ_0086913, and hsa_circ_0065188) and two lncRNAs (SNHG20 and LINC00582) were selected for future study. The expression levels of circRNAs hsa_circ_0049170 and hsa_circ_0087059 were found to be upregulated in LPS-induced BEAS-2B cells (Fig. 8C); the expression level of hsa_circ_0086913 was upregulated and that of hsa_circ_0065188 was downregulated in LPS-induced HUVECs (Fig. 8C). Meanwhile, the expression level of lncRNA SNHG20 was upregulated and that of LINC00582 was downregulated in LPS-induced BEAS-2B cells (Fig. 8C). These results were consistent with the results of exosomal circRNA and lncRNA profiles by microarray analysis of UCB in the BPD and NBPD groups.

## Discussion

Exosomal circRNAs and lncRNAs attract increasing attention owing to their high regulatory potential [25–27]. Previous studies have examined UCB-derived exosomes from patients with preeclampsia and GDM [18, 19] because of their distant regulatory potency. Further, exosomal circRNAs and lncRNAs may carry important information and play roles while in cells, belonging to exosomes released from synaptoneurosomes [28–32]. These studies indicated exosomal circRNAs and lncRNAs could be used as molecular markers for clinically diagnosing and treating various pathologies [33, 34]. In the present study, exosomal circRNAs, lncRNAs, and mRNAs in UCB were significantly altered in infants with BPD. In all, 317 circRNAs, 104 lncRNAs, and 135 mRNAs were differentially expressed in UCB-derived exosomes of infants with BPD compared with those with NBPD.

GO and KEGG analyses were carried out to further examine the roles of the differentially expressed exosomal circRNAs, lncRNAs, and mRNAs. As shown earlier, the most enriched GO terms and KEGG pathways of exosomal RNAs were associated with endothelial or epithelial cell development, including “angiogenesis” [35], “mammalian target of rapamycin (mTOR) signaling pathway” [36], “Wnt signaling pathway” [37, 38], “Epidermal growth factor receptor tyrosine kinase inhibitor resistance” [39], and “transforming growth factor-beta receptor signaling” [40]. Meanwhile, several GO terms and pathways connected with exosome transport were also significantly enriched, including “regulation of vesicle-mediated transport,” “vacuolar acidification,” and “extracellular matrix (ECM)–receptor interaction.” The aforementioned results showed that KEGG terms for differentially expressed exosomal circRNAs, lncRNAs, and mRNAs with most enriched genes were “transport and catabolism,” “signal transduction,” “translation,” “infectious diseases: viral,” “immune system,” and “carbohydrate metabolism.” These results suggested that BPD development involved complex and diverse pathophysiological events.

Among them, the “mTOR signaling pathway” and the identified coding genes have gained interest because of their high enrichment factor and large gene number. mTOR is known to have two protein complexes with distinct functions, which are complex 1 (mTORC1) and complex 2 (mTORC2) [41]. mTORC1 comprises mTOR, MTOR-associated protein, LST8 homolog (mLST8), raptor, and two repressors (PRAS40 and DEPTOR). Recent data revealed that hyperoxia exposure of murine and baboon lungs in BPD is associated with insufficient activation of 5′-adenosine monophosphate-activated protein kinase and mTORC1 hyperactivity [42]. In vitro, mTOR inhibition significantly promotes the proliferation of basal cells derived from neonatal tracheal aspirate, which may constitute a critical model system for studying late fetal lung development and perinatal lung diseases, including BPD [36]. Another study found that inhibition of regulatory-associated protein of mTOR, a major subunit of mTORC1, prevented hyperoxia-stimulated lung damage by heightening autophagy and weakening apoptotic death in newborn mice [43]. These results and the aforementioned findings suggested that differentially expressed exosomal RNAs might play a crucial role in BPD through the mTOR signaling pathway.

At present, many pathways related to BPD exist. Interestingly, in this study, “ECM–receptor interaction” was found, which is rarely studied in BPD and has been found in both lncRNA and circRNA KEGG analysis results. The ECM is a noncellular three-dimensional network polymer consisting of fibronectin, elastin, collagens, proteoglycans, and several other glycoproteins [44]. Damaged ECM is known to provoke apoptosis of overlying pulmonary epithelial cells and alveolar unit loss associated with BPD [45, 46]. Further, exosomes are part of the ECM and are involved in the remodeling of the extracellular environment [7]. Exosomes from mesenchymal stem cells (MSCs) could reverse alveolar injury and septal thickness associated with hyperoxia-dependent lung damage in mice with experimental BPD [15]. Meanwhile, in BPD lung secretions, pathogenic exosomes are also detected, and hyperoxia and microbial products could induce the abnormal expression of exosomal miRNAs [12, 47]. In addition, numerous reports have demonstrated that exosomes regulate vascular growth, proliferation, metastasis, and apoptosis through exosomal circRNAs and lncRNAs [48–51]. These results suggested that ECM–receptor interactions and enriched circRNAs and lncRNAs might be pivotal in the role of exosomes in BPD progression.

In this study, lung epithelial cells BEAS-2B and HUVECs were used as research objects for future analyses to verify the differentially expressed RNAs. Excessive inflammation persists in infants with BPD, which could be caused by a variety of factors, including mechanical ventilation and infection. Therefore, the inflammatory response of BPD by LPS was reflected in this study. Further, studies have reported that BEAS-2B cells and HUVECs could be stimulated by LPS to induce inflammatory response [52–54], consistent with the results of this study. Then, the expression levels of differentially expressed circRNAs and lncRNAs in LPS-induced BEAS-2B cells and HUVECs were explored. The results of qRT-PCR analyses showed similar change trends as in UCB-derived exosomes of preterm infants with BPD. These results suggested that these differentially expressed RNAs may play a potential role in BPD, which are worthy of deeper functional studies.

The molecular functions of differentially expressed exosomal circRNAs were studied by exploring them from the perspective of ceRNA. In this study, 13 of the top 10 upregulated and downregulated expression of circRNAs showed binding sites for miRNAs, of which some were connected with pulmonary diseases. For instance, miR-1207-5p, miR-608, and miR-4640-5p were reported to be related to non-small-cell lung cancer [55–58]. According to the PCC, fold change, and expression level, the possible role of exosomal hsa_circ_0086913 was explored, which was 3.60-fold upregulated in UCB-derived exosomes from the BPD group and LPS-induced HUVECs. The ceRNA analysis revealed that hsa-miR-330-5p, hsa-miR-4656, hsa-miR-6829-3p, hsa-miR-103a-3p, hsa-miR-107, hsa-miR-4688, hsa-miR-7161-3p, hsa-miR-3192-5p, hsa-miR-3620-5p, hsa-miR-4656, hsa-miR-1182, hsa-miR-4656, hsa-miR-4688, and hsa-miR-6783-3p potentially interacted with hsa_circ_0086913. Among them, hsa_circ_0086913/hsa-miR-103a-3p/transmembrane 4 L six family member 1 (TM4SF1) was predicted with a PCC of 0.93. In a recent study, the researchers found that the expression level of hsa-miR-103a-3p related to the phosphatidylinositol 3-kinase/protein kinase B (PI3K/Akt) signaling pathway was decreased in UCB-derived exosomes from the BPD group compared with the NBPD group [16]. Further, the overexpression of hsa-miR-103a-3p in normal HUVECs significantly promoted cell proliferation, cell migration, and tube formation [16]. In addition, the expression level of TM4SF1, a potential target gene of hsa-miR-103a-3p, was upregulated in UCB-derived exosomes of infants with BPD. It has been found to promote angiogenesis via the Akt signaling pathway [59]. These results demonstrated that UCB-derived exosomal hsa_circ_0086913 of infants with BPD might contribute to the development of BPD, possibly via the interaction network hsa_circ_0086913/hsa-miR-103a-3p/TM4SF1.

Meanwhile, in this study, most of the exosomal lncRNAs were found to have predicted target miRNAs that may be related to BPD. For instance, a total of 27 miRNAs were found to potentially match with upregulated lncRNA SNHG20 (also referred to as ENST00000566583); of them, the interaction network lncRNA-SNHG20/hsa-miR-6720-5p/spermine synthase was predicted with the highest PCC of 0.99. Previous studies reported that the knockdown of SNHG20 inhibited cell proliferation and invasion in human lung epithelial cells A549 cells [60–62]. In addition, the expression level of lncRNA SNHG20 has been shown to be significantly upregulated in hepatocellular carcinoma and could promote the epithelial-to-mesenchymal transition (EMT) [63]. The expression level of lncRNA SNHG20 was upregulated in UCB-derived exosomes of infants with BPD as well as in LPS-induced BEAS-2B cells. EMT is a process of conversion of epithelial cells into mesenchymal cells and occurs in long-term survivors with BPD [64, 65]. Therefore, in this study, it was predicted that lncRNA SNHG20 might promote EMT in BPD, which is worthy of future studies.

In summary, exosomal circRNA, lncRNA, and mRNA profiles in the UCB of newborns with BPD were determined, and 317 circRNAs, 104 lncRNAs, and 135 mRNAs were found to be significantly altered. The aforementioned findings indicated that exosomal circRNAs/lncRNAs might have vital roles in BPD pathogenesis. Through bioinformatics, several potential exosomal circRNA/lncRNA–miRNA–mRNA networks were also successfully constructed, which might be involved in BPD. Two differentially expressed circRNAs (hsa_circ_0049170, hsa_circ_0087059) and two lncRNAs (SNHG20 and LINC00582) were identified in LPS-induced BEAS-2B cells, and two other circRNAs (hsa_circ_0086913 and hsa_circ_0065188) were also identified in LPS-induced HUVECs. These results provided a sound basis for further investigations assessing the potential biological functions of exosomal circRNAs and lncRNAs in BPD.

## Supplementary Information

Below is the link to the electronic supplementary material.

Supplementary file1 (TIF 8969 KB)

Supplementary file2 (DOCX 16 KB)

Supplementary file3 (DOCX 34 KB)

Supplementary file4 (DOCX 41 KB)

## Data Availability

Relevant data has been uploaded to GEO repository and is scheduled to be released on Dec. 31, 2023. The access number is GSE190215 (https://www.ncbi.nlm.nih.gov/geo/query/acc.cgi?acc=GSE190215).

## Abbreviations

BPD: Bronchopulmonary dysplasia
BW: Birthweight
CCK-8: Cell counting kit-8
circRNAs: Circular RNAs
CPAP: Continuous positive airway pressure
EOS: Early-onset neonatal sepsis
ECM: Extracellular matrix
EV: Extracellular vesicle
GO: Gene Ontology
GA: Gestational age
GDM: Gestational diabetes mellitus
HUVECs: Human umbilical vein endothelial cells
IL: Interleukin
IMV: Invasive mechanical ventilation
IVH: Intraventricular hemorrhage
KEGG: Kyoto Encyclopedia of Genes and Genomes
LOS: Late-onset neonatal sepsis
LPS: Lipopolysaccharide
mTOR: Mammalian target of rapamycin
MSCs: Mesenchymal stem cells
miRNA: MicroRNA
MREs: MiRNA response elements
NTA: Nanoparticle tracking analysis
NEC: Necrotizing enterocolitis
PDA: Patent ductus arteriosus
PCC: Pearson correlation coefficient
PBS: Phosphate-buffered saline
PS: Pulmonary surfactant
PVDF: Polyvinylidene fluoride
PROM: Premature rupture of membranes
qPCR: Quantitative polymerase chain reaction
RDS: Respiratory distress syndrome
ROP: Retinopathy of prematurity
SNHG20: Small nucleolar RNA host gene 20
TM4SF1: Transmembrane 4 L six family member 1
TEM: Transmission electron microscopy
TNF: Tumor necrosis factor
UCB: Umbilical cord blood
