# Supplementary figures and images for: CircRNA, lncRNA, and mRNA profiles of umbilical cord blood exosomes from preterm newborns showing bronchopulmonary dysplasia
Source: Eur J Pediatr. 2022 Jul 5;181(9):3345–65. doi: 10.1007/s00431-022-04544-2 (PMC9395505; doi:10.1007/s00431-022-04544-2)

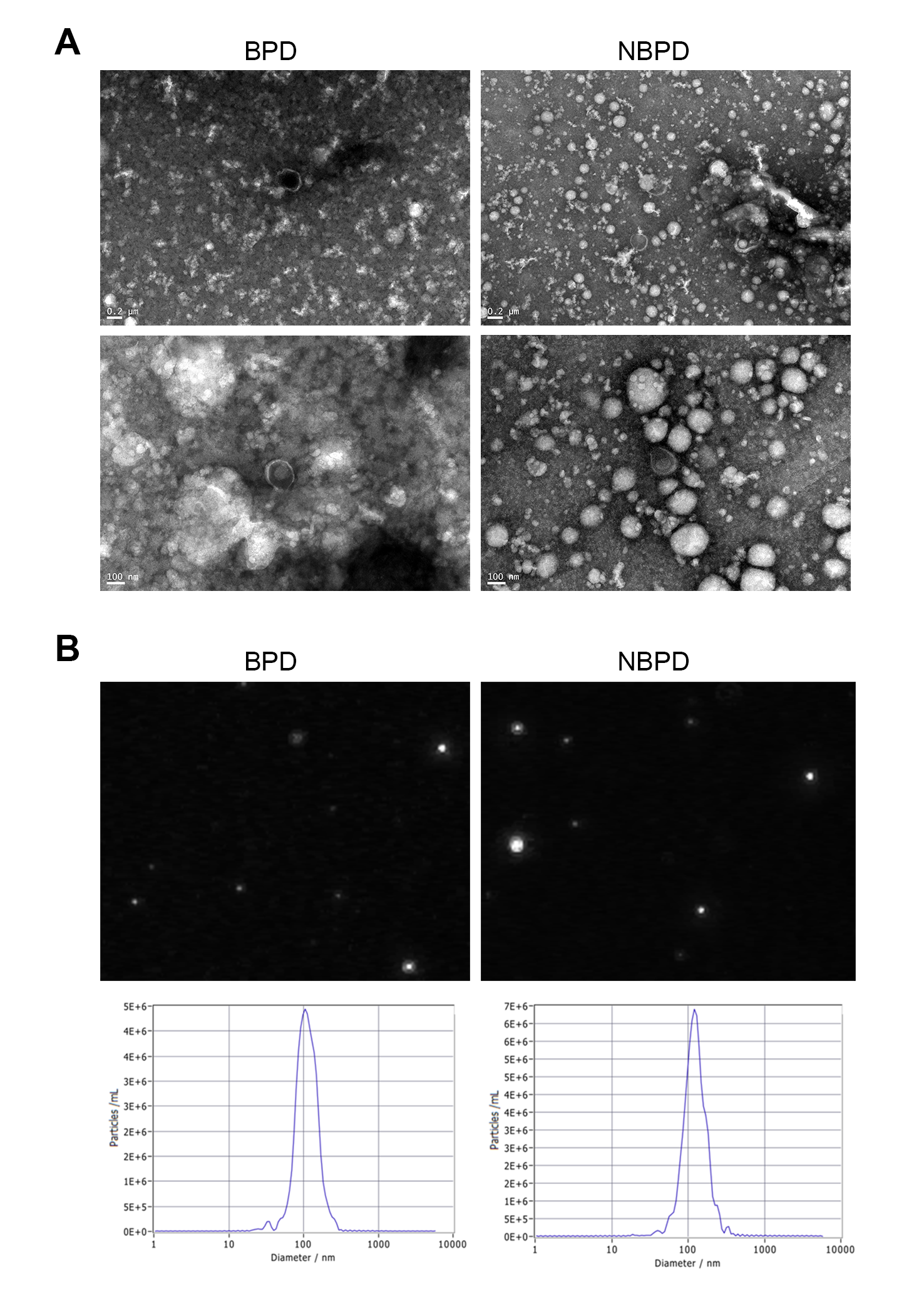

Supplement: Supplementary file 1 — Supplementary file1 (TIF 8969 KB) [file 431_2022_4544_MOESM1_ESM.tif]
