# Supplementary material for: CircRNA, lncRNA, and mRNA profiles of umbilical cord blood exosomes from preterm newborns showing bronchopulmonary dysplasia
Source: Eur J Pediatr. 2022 Jul 5;181(9):3345–65. doi: 10.1007/s00431-022-04544-2 (PMC9395505; doi:10.1007/s00431-022-04544-2)
Supplement: Supplementary file 2 — Supplementary file2 (DOCX 16 KB) [file 431_2022_4544_MOESM2_ESM.docx]

| **Supplementary Table S1 Primer sequences** | |
| --- | --- |
| Gene name | Primer sequences |
| CircRNA hsa_circ_0086913 | F: 5'-CTGCTGAAAATGAAGAGGCTCA-3' |
|  | R: 5'-TGCTGCCTTCACTTCCTGTA-3' |
| CircRNA hsa_circ_0007372 | F: 5'-GCTTTTGGGCAAGGACTTCC-3' |
|  | R: 5'-GGTCCATGTCTTTGCCTCTG-3' |
| CircRNA hsa_circ_0065188 | F: 5'-TTCATTACCTGCAGAGTCGG-3' |
|  | R: 5'-ACTTGCGGAGGACACTACAG-3' |
| CircRNA hsa_circ_0049170 | F: 5'-ATTCAGGAGGAGATGGGTGC-3' |
|  | R: 5'-TCCCGACCCCAGCTTCTG-3' |
| CircRNA hsa_circ_0087059 | F: 5'-AGTGAAGAATGGTGGCTGGA-3' |
|  | R: 5'-TCCACCTCACAGCTATGCG-3' |
| lncRNA MAGI2-AS3 | F: 5'-CGCCTGGGTGTGTGTTGTTA-3' |
|  | R: 5'-CTGGACTTAAGCTGGGCACA-3' |
| lncRNA BASP1-AS1 | F: 5'-CCTGGCTCCACATACAGCAA-3' |
|  | R: 5'-TCACCATGTTAGCCAGGCTG-3' |
| lncRNA SLC2A1-AS1 | F: 5'-ATTCGCTGTGTGACTCAGGG-3' |
|  | R: 5'-CATTTTCTGCTGTGTGGGGC-3' |
| lncRNA SNHG20 | F: 5'-AATATCCCCCGACGATTGGC-3' |
|  | R: 5'-GGGAGCAGGAAGGCATCTTT-3' |
| lncRNA LINC00582 | F: 5'-AGGCGGAAAGAACTACGTGA-3' |
|  | R: 5'-TCACACCCAGACTCTGCATT-3' |
| GAPDH | F: 5'-GAAGGTGAAGGTCGGAGTC-3' |
|  | R: 5'-GAAGATGGTGATGGGATTTC-3' |
