# Supplementary material for: CircRNA, lncRNA, and mRNA profiles of umbilical cord blood exosomes from preterm newborns showing bronchopulmonary dysplasia
Source: Eur J Pediatr. 2022 Jul 5;181(9):3345–65. doi: 10.1007/s00431-022-04544-2 (PMC9395505; doi:10.1007/s00431-022-04544-2)
Supplement: Supplementary file 3 — Supplementary file3 (DOCX 34 KB) [file 431_2022_4544_MOESM3_ESM.docx]

| **Supplementary Table S2 CircRNA-miRNA-mRNA regulations in network** | | | | |
| --- | --- | --- | --- | --- |
| mRNA | circRNA | miRNA | PCC | -log10 *p*-value |
| KLC2 | hsa_circ_0084333 | hsa-miR-3127-3p | 0.99 | 5.20 |
| KLC2 | hsa_circ_0084333 | hsa-miR-6765-5p | 0.99 | 5.20 |
| TPO | hsa_circ_0081368 | hsa-miR-4691-3p | 0.99 | 5.16 |
| MEN1 | hsa_circ_0086018 | hsa-miR-1207-5p | 0.98 | 4.69 |
| MEN1 | hsa_circ_0086018 | hsa-miR-4685-5p | 0.98 | 4.69 |
| MEN1 | hsa_circ_0086018 | hsa-miR-744-5p | 0.98 | 4.69 |
| MEN1 | hsa_circ_0086018 | hsa-miR-762 | 0.98 | 4.69 |
| BHLHA9 | hsa_circ_0045828 | hsa-miR-4739 | 0.98 | 4.42 |
| TNXB | hsa_circ_0118639 | hsa-miR-5193 | 0.97 | 4.34 |
| PDZD4 | hsa_circ_0081368 | hsa-miR-145-5p | 0.97 | 4.34 |
| PDZD4 | hsa_circ_0081368 | hsa-miR-4749-5p | 0.97 | 4.34 |
| PDZD4 | hsa_circ_0081368 | hsa-miR-6793-3p | 0.97 | 4.34 |
| LRRC31 | hsa_circ_0045828 | hsa-miR-6788-5p | 0.97 | 4.19 |
| KLC2 | hsa_circ_0086018 | hsa-miR-1207-5p | 0.97 | 4.18 |
| KLC2 | hsa_circ_0086018 | hsa-miR-149-5p | 0.97 | 4.18 |
| KLC2 | hsa_circ_0086018 | hsa-miR-4640-5p | 0.97 | 4.18 |
| KLC2 | hsa_circ_0086018 | hsa-miR-4685-5p | 0.97 | 4.18 |
| KLC2 | hsa_circ_0086018 | hsa-miR-6751-5p | 0.97 | 4.18 |
| KLC2 | hsa_circ_0086018 | hsa-miR-6803-5p | 0.97 | 4.18 |
| KLC2 | hsa_circ_0086018 | hsa-miR-6812-5p | 0.97 | 4.18 |
| KLC2 | hsa_circ_0086018 | hsa-miR-6836-5p | 0.97 | 4.18 |
| KLC2 | hsa_circ_0086018 | hsa-miR-7155-5p | 0.97 | 4.18 |
| RILPL2 | hsa_circ_0061761 | hsa-miR-4691-3p | 0.96 | 3.89 |
| KIFC3 | hsa_circ_0045828 | hsa-miR-5193 | 0.96 | 3.86 |
| KIFC3 | hsa_circ_0045828 | hsa-miR-6810-5p | 0.96 | 3.86 |
| TIGD5 | hsa_circ_0007372 | hsa-miR-6722-3p | 0.96 | 3.84 |
| MYCBP2 | hsa_circ_0045828 | hsa-miR-6861-5p | 0.96 | 3.81 |
| MYCBP2 | hsa_circ_0045828 | hsa-miR-6884-5p | 0.96 | 3.81 |
| MYCBP2 | hsa_circ_0045828 | hsa-miR-8073 | 0.96 | 3.81 |
| NPNT | hsa_circ_0045828 | hsa-miR-4739 | 0.96 | 3.80 |
| APBB1 | hsa_circ_0045828 | hsa-miR-3192-5p | 0.96 | 3.80 |
| APBB1 | hsa_circ_0045828 | hsa-miR-5193 | 0.96 | 3.80 |
| TTLL9 | hsa_circ_0045828 | hsa-miR-8073 | 0.96 | 3.73 |
| SYMPK | hsa_circ_0086913 | hsa-miR-330-5p | 0.96 | 3.72 |
| SYMPK | hsa_circ_0086913 | hsa-miR-4656 | 0.96 | 3.72 |
| SYMPK | hsa_circ_0086913 | hsa-miR-6829-3p | 0.96 | 3.72 |
| APP | hsa_circ_0073391 | hsa-miR-3064-5p | 0.96 | 3.66 |
| APP | hsa_circ_0073391 | hsa-miR-6787-5p | 0.96 | 3.66 |
| TTLL9 | hsa_circ_0081368 | hsa-miR-3667-3p | 0.95 | 3.63 |
| TTLL9 | hsa_circ_0081368 | hsa-miR-4685-3p | 0.95 | 3.63 |
| PCYT2 | hsa_circ_0081368 | hsa-miR-548b-3p | 0.95 | 3.61 |
| PCYT2 | hsa_circ_0081368 | hsa-miR-6889-5p | 0.95 | 3.61 |
| MYCBP2 | hsa_circ_0081368 | hsa-miR-520f-3p | 0.95 | 3.61 |
| MYCBP2 | hsa_circ_0081368 | hsa-miR-766-3p | 0.95 | 3.61 |
| TRAF4 | hsa_circ_0081368 | hsa-miR-1343-3p | 0.95 | 3.56 |
| TRAF4 | hsa_circ_0081368 | hsa-miR-6857-3p | 0.95 | 3.56 |
| KRTAP10-3 | hsa_circ_0037782 | hsa-miR-4722-5p | 0.95 | 3.55 |
| TPO | hsa_circ_0007372 | hsa-miR-6722-3p | 0.95 | 3.43 |
| PXN | hsa_circ_0007372 | hsa-miR-6846-5p | 0.94 | 3.40 |
| DNAJA3 | hsa_circ_0045828 | hsa-miR-5193 | 0.94 | 3.39 |
| FANCC | hsa_circ_0045828 | hsa-miR-3192-5p | 0.94 | 3.36 |
| MEN1 | hsa_circ_0045828 | hsa-miR-3192-5p | 0.94 | 3.35 |
| MEN1 | hsa_circ_0045828 | hsa-miR-6089 | 0.94 | 3.35 |
| SBK3 | hsa_circ_0086018 | hsa-miR-4685-5p | 0.94 | 3.35 |
| SBK3 | hsa_circ_0086018 | hsa-miR-6891-3p | 0.94 | 3.35 |
| SBK3 | hsa_circ_0086018 | hsa-miR-762 | 0.94 | 3.35 |
| HAGHL | hsa_circ_0065188 | hsa-miR-2277-3p | 0.94 | 3.34 |
| NKAIN1 | hsa_circ_0086018 | hsa-miR-4640-5p | 0.94 | 3.33 |
| NKAIN1 | hsa_circ_0086018 | hsa-miR-608 | 0.94 | 3.33 |
| NKAIN1 | hsa_circ_0086018 | hsa-miR-6756-5p | 0.94 | 3.33 |
| NKAIN1 | hsa_circ_0086018 | hsa-miR-6812-5p | 0.94 | 3.33 |
| NKAIN1 | hsa_circ_0086018 | hsa-miR-744-5p | 0.94 | 3.33 |
| NKAIN1 | hsa_circ_0086018 | hsa-miR-762 | 0.94 | 3.33 |
| MEN1 | hsa_circ_0078460 | hsa-miR-6744-5p | 0.94 | 3.32 |
| MEN1 | hsa_circ_0084333 | hsa-miR-3620-5p | 0.94 | 3.30 |
| MEN1 | hsa_circ_0084333 | hsa-miR-4505 | 0.94 | 3.30 |
| MEN1 | hsa_circ_0084333 | hsa-miR-5001-5p | 0.94 | 3.30 |
| MEN1 | hsa_circ_0084333 | hsa-miR-762 | 0.94 | 3.30 |
| DNAJC13 | hsa_circ_0078460 | hsa-miR-6509-3p | 0.94 | 3.29 |
| DNAJC22 | hsa_circ_0037782 | hsa-miR-212-5p | 0.94 | 3.28 |
| DNAJC22 | hsa_circ_0037782 | hsa-miR-4722-5p | 0.94 | 3.28 |
| DNAJC22 | hsa_circ_0037782 | hsa-miR-7107-5p | 0.94 | 3.28 |
| ZNF574 | hsa_circ_0081368 | hsa-miR-93-5p | 0.93 | 3.17 |
| APBB1 | hsa_circ_0007372 | hsa-miR-6873-3p | 0.93 | 3.17 |
| PDZD4 | hsa_circ_0045828 | hsa-miR-2392 | 0.93 | 3.16 |
| PDZD4 | hsa_circ_0045828 | hsa-miR-4268 | 0.93 | 3.16 |
| PDZD4 | hsa_circ_0045828 | hsa-miR-5193 | 0.93 | 3.16 |
| PDZD4 | hsa_circ_0045828 | hsa-miR-6089 | 0.93 | 3.16 |
| NSMF | hsa_circ_0084333 | hsa-miR-1587 | 0.93 | 3.12 |
| NSMF | hsa_circ_0084333 | hsa-miR-3620-5p | 0.93 | 3.12 |
| NSMF | hsa_circ_0084333 | hsa-miR-762 | 0.93 | 3.12 |
| NPNT | hsa_circ_0007372 | hsa-miR-6722-3p | 0.93 | 3.10 |
| NSMF | hsa_circ_0086018 | hsa-miR-1207-5p | 0.93 | 3.09 |
| NSMF | hsa_circ_0086018 | hsa-miR-4640-5p | 0.93 | 3.09 |
| NSMF | hsa_circ_0086018 | hsa-miR-4685-5p | 0.93 | 3.09 |
| NSMF | hsa_circ_0086018 | hsa-miR-4717-5p | 0.93 | 3.09 |
| NSMF | hsa_circ_0086018 | hsa-miR-4728-5p | 0.93 | 3.09 |
| NSMF | hsa_circ_0086018 | hsa-miR-608 | 0.93 | 3.09 |
| NSMF | hsa_circ_0086018 | hsa-miR-6751-5p | 0.93 | 3.09 |
| NSMF | hsa_circ_0086018 | hsa-miR-6756-5p | 0.93 | 3.09 |
| NSMF | hsa_circ_0086018 | hsa-miR-6803-5p | 0.93 | 3.09 |
| NSMF | hsa_circ_0086018 | hsa-miR-6806-5p | 0.93 | 3.09 |
| NSMF | hsa_circ_0086018 | hsa-miR-762 | 0.93 | 3.09 |
| PAX7 | hsa_circ_0086018 | hsa-miR-149-5p | 0.93 | 3.08 |
| PAX7 | hsa_circ_0086018 | hsa-miR-4268 | 0.93 | 3.08 |
| PAX7 | hsa_circ_0086018 | hsa-miR-4685-5p | 0.93 | 3.08 |
| PAX7 | hsa_circ_0086018 | hsa-miR-6751-5p | 0.93 | 3.08 |
| PAX7 | hsa_circ_0086018 | hsa-miR-6812-5p | 0.93 | 3.08 |
| PAX7 | hsa_circ_0086018 | hsa-miR-762 | 0.93 | 3.08 |
| CRTC1 | hsa_circ_0037782 | hsa-miR-4722-5p | 0.93 | 3.08 |
| NPNT | hsa_circ_0086018 | hsa-miR-608 | 0.93 | 3.06 |
| TM4SF1 | hsa_circ_0086913 | hsa-miR-103a-3p | 0.93 | 3.06 |
| TM4SF1 | hsa_circ_0086913 | hsa-miR-107 | 0.93 | 3.06 |
| FERMT3 | hsa_circ_0086913 | hsa-miR-4688 | 0.93 | 3.04 |
| PAX7 | hsa_circ_0007372 | hsa-miR-6846-5p | 0.93 | 3.03 |
| DNAJC22 | hsa_circ_0081368 | hsa-miR-93-5p | 0.93 | 3.02 |
| MGP | hsa_circ_0061761 | hsa-miR-3934-5p | 0.93 | 3.00 |
| FERMT3 | hsa_circ_0073391 | hsa-miR-3147 | 0.92 | 2.96 |
| FERMT3 | hsa_circ_0073391 | hsa-miR-6893-3p | 0.92 | 2.96 |
| TIGD5 | hsa_circ_0086018 | hsa-miR-1207-5p | 0.92 | 2.95 |
| TIGD5 | hsa_circ_0086018 | hsa-miR-4685-5p | 0.92 | 2.95 |
| TIGD5 | hsa_circ_0086018 | hsa-miR-7155-5p | 0.92 | 2.95 |
| TIGD5 | hsa_circ_0086018 | hsa-miR-7156-3p | 0.92 | 2.95 |
| TIGD5 | hsa_circ_0086018 | hsa-miR-762 | 0.92 | 2.95 |
| DNAJC22 | hsa_circ_0045828 | hsa-miR-8073 | 0.92 | 2.94 |
| DUS2 | hsa_circ_0081368 | hsa-miR-766-3p | 0.92 | 2.94 |
| MYCBP2 | hsa_circ_0086018 | hsa-miR-2861 | 0.92 | 2.93 |
| MYCBP2 | hsa_circ_0086018 | hsa-miR-4685-5p | 0.92 | 2.93 |
| MYCBP2 | hsa_circ_0086018 | hsa-miR-6806-5p | 0.92 | 2.93 |
| PAX7 | hsa_circ_0045828 | hsa-miR-4268 | 0.92 | 2.92 |
| PAX7 | hsa_circ_0045828 | hsa-miR-4739 | 0.92 | 2.92 |
| PAX7 | hsa_circ_0045828 | hsa-miR-6788-5p | 0.92 | 2.92 |
| PAX7 | hsa_circ_0045828 | hsa-miR-6861-5p | 0.92 | 2.92 |
| PXN | hsa_circ_0045828 | hsa-miR-4739 | 0.92 | 2.91 |
| EMD | hsa_circ_0086913 | hsa-miR-7161-3p | 0.92 | 2.89 |
| SYMPK | hsa_circ_0073391 | hsa-miR-15a-5p | 0.92 | 2.89 |
| SYMPK | hsa_circ_0073391 | hsa-miR-6749-5p | 0.92 | 2.89 |
| SYMPK | hsa_circ_0073391 | hsa-miR-6765-5p | 0.92 | 2.89 |
| SYMPK | hsa_circ_0073391 | hsa-miR-6893-3p | 0.92 | 2.89 |
| PAX7 | hsa_circ_0081368 | hsa-miR-326 | 0.92 | 2.89 |
| PAX7 | hsa_circ_0081368 | hsa-miR-4685-3p | 0.92 | 2.89 |
| FPGS | hsa_circ_0045828 | hsa-miR-130b-5p | 0.92 | 2.88 |
| VPS37D | hsa_circ_0086018 | hsa-miR-4640-5p | 0.92 | 2.88 |
| VPS37D | hsa_circ_0086018 | hsa-miR-4685-5p | 0.92 | 2.88 |
| VPS37D | hsa_circ_0086018 | hsa-miR-762 | 0.92 | 2.88 |
| PCYT2 | hsa_circ_0007372 | hsa-miR-6722-3p | 0.92 | 2.87 |
| NKAIN1 | hsa_circ_0081368 | hsa-miR-1976 | 0.92 | 2.87 |
| NKAIN1 | hsa_circ_0081368 | hsa-miR-6889-5p | 0.92 | 2.87 |
| PLB1 | hsa_circ_0037782 | hsa-miR-212-5p | 0.91 | 2.84 |
| TPO | hsa_circ_0086018 | hsa-miR-1207-5p | 0.91 | 2.82 |
| TPO | hsa_circ_0086018 | hsa-miR-608 | 0.91 | 2.82 |
| NPNT | hsa_circ_0037782 | hsa-miR-6740-3p | 0.91 | 2.78 |
| TRAF4 | hsa_circ_0045828 | hsa-miR-4739 | 0.91 | 2.78 |
| APBB1 | hsa_circ_0086018 | hsa-miR-149-5p | 0.91 | 2.77 |
| APBB1 | hsa_circ_0086018 | hsa-miR-6836-5p | 0.91 | 2.77 |
| APBB1 | hsa_circ_0086018 | hsa-miR-6891-3p | 0.91 | 2.77 |
| FAM189B | hsa_circ_0073391 | hsa-miR-1908-5p | 0.91 | 2.76 |
| FAM189B | hsa_circ_0073391 | hsa-miR-4739 | 0.91 | 2.76 |
| FAM189B | hsa_circ_0073391 | hsa-miR-6754-5p | 0.91 | 2.76 |
| FAM189B | hsa_circ_0073391 | hsa-miR-6893-3p | 0.91 | 2.76 |
| FAM189B | hsa_circ_0073391 | hsa-miR-7111-3p | 0.91 | 2.76 |
| PXN | hsa_circ_0086018 | hsa-miR-1207-5p | 0.91 | 2.76 |
| PXN | hsa_circ_0086018 | hsa-miR-149-5p | 0.91 | 2.76 |
| PXN | hsa_circ_0086018 | hsa-miR-6511a-5p | 0.91 | 2.76 |
| PXN | hsa_circ_0086018 | hsa-miR-6756-5p | 0.91 | 2.76 |
| PXN | hsa_circ_0086018 | hsa-miR-6780b-5p | 0.91 | 2.76 |
| PXN | hsa_circ_0086018 | hsa-miR-6836-5p | 0.91 | 2.76 |
| PXN | hsa_circ_0086018 | hsa-miR-762 | 0.91 | 2.76 |
| QSOX1 | hsa_circ_0118639 | hsa-miR-4421 | 0.91 | 2.75 |
| QSOX1 | hsa_circ_0118639 | hsa-miR-4731-5p | 0.91 | 2.75 |
| DNAJC13 | hsa_circ_0086018 | hsa-miR-6812-5p | 0.91 | 2.72 |
| SMIM1 | hsa_circ_0086913 | hsa-miR-3192-5p | 0.91 | 2.71 |
| SMIM1 | hsa_circ_0086913 | hsa-miR-3620-5p | 0.91 | 2.71 |
| SMIM1 | hsa_circ_0086913 | hsa-miR-4656 | 0.91 | 2.71 |
| DNAJC13 | hsa_circ_0081368 | hsa-miR-145-5p | 0.91 | 2.70 |
| DNAJC13 | hsa_circ_0081368 | hsa-miR-766-3p | 0.91 | 2.70 |
| PRSS3 | hsa_circ_0034846 | hsa-miR-541-3p | 0.90 | 2.70 |
| SGTA | hsa_circ_0037782 | hsa-miR-4524b-3p | 0.90 | 2.70 |
| SGTA | hsa_circ_0037782 | hsa-miR-6740-3p | 0.90 | 2.70 |
| NSMF | hsa_circ_0034846 | hsa-miR-874-5p | 0.90 | 2.69 |
| NKAIN1 | hsa_circ_0045828 | hsa-miR-6788-5p | 0.90 | 2.69 |
| ZER1 | hsa_circ_0086913 | hsa-miR-1182 | 0.90 | 2.67 |
| ZER1 | hsa_circ_0086913 | hsa-miR-4656 | 0.90 | 2.67 |
| ZER1 | hsa_circ_0086913 | hsa-miR-4688 | 0.90 | 2.67 |
| ZER1 | hsa_circ_0086913 | hsa-miR-6783-3p | 0.90 | 2.67 |
| RAB44 | hsa_circ_0045828 | hsa-miR-1273h-5p | 0.90 | 2.66 |
| RAB44 | hsa_circ_0045828 | hsa-miR-5193 | 0.90 | 2.66 |
| RAB44 | hsa_circ_0045828 | hsa-miR-6780a-5p | 0.90 | 2.66 |
| RAB44 | hsa_circ_0045828 | hsa-miR-6868-3p | 0.90 | 2.66 |
| TM4SF1 | hsa_circ_0073391 | hsa-miR-4446-3p | 0.90 | 2.66 |
| ZNF574 | hsa_circ_0084333 | hsa-miR-6081 | 0.90 | 2.66 |
| KIFC3 | hsa_circ_0007372 | hsa-miR-6846-5p | 0.90 | 2.65 |
| TNXB | hsa_circ_0013996 | hsa-miR-3616-3p | 0.90 | 2.65 |
| TNXB | hsa_circ_0013996 | hsa-miR-378a-3p | 0.90 | 2.65 |
| TNXB | hsa_circ_0013996 | hsa-miR-378c | 0.90 | 2.65 |
| TNXB | hsa_circ_0013996 | hsa-miR-378h | 0.90 | 2.65 |
| TIGD5 | hsa_circ_0037782 | hsa-miR-370-3p | 0.90 | 2.64 |
