# Supplementary material for: CircRNA, lncRNA, and mRNA profiles of umbilical cord blood exosomes from preterm newborns showing bronchopulmonary dysplasia
Source: Eur J Pediatr. 2022 Jul 5;181(9):3345–65. doi: 10.1007/s00431-022-04544-2 (PMC9395505; doi:10.1007/s00431-022-04544-2)
Supplement: Supplementary file 4 — Supplementary file4 (DOCX 41 KB) [file 431_2022_4544_MOESM4_ESM.docx]

| **Supplementary Table S3 LncRNA-miRNA-mRNA regulations in network** | | | | |
| --- | --- | --- | --- | --- |
| mRNA | lncRNA | miRNA | PCC | -log10 *p*-value |
| SMS | ENST00000566583 | hsa-miR-6720-5p | 0.99 | 5.61 |
| DNAJA3 | ENST00000661332 | hsa-miR-6766-5p | 0.98 | 5.05 |
| SMS | ENST00000434411 | hsa-miR-6720-5p | 0.98 | 5.00 |
| PARP8 | ENST00000434411 | hsa-miR-6791-5p | 0.98 | 4.94 |
| LRRTM1 | ENST00000661332 | hsa-miR-3190-3p | 0.98 | 4.67 |
| PARP8 | ENST00000566583 | hsa-miR-6791-5p | 0.98 | 4.67 |
| RILPL2 | ENST00000434411 | hsa-miR-6791-5p | 0.98 | 4.66 |
| CAMP | ENST00000456953 | hsa-miR-1343-3p | 0.98 | 4.51 |
| PLB1 | ENST00000448058 | hsa-miR-3064-5p | 0.98 | 4.48 |
| BHLHA9 | ENST00000661332 | hsa-miR-6754-5p | 0.97 | 4.33 |
| ABCC10 | ENST00000555433 | hsa-miR-3116 | 0.97 | 4.31 |
| PDZD4 | ENST00000502301 | hsa-miR-6860 | 0.97 | 4.27 |
| MYCBP2 | ENST00000553812 | hsa-miR-6858-5p | 0.97 | 4.27 |
| HOMER3 | ENST00000430373 | hsa-miR-6856-5p | 0.97 | 4.24 |
| DUSP1 | ENST00000430373 | hsa-miR-6511a-5p | 0.97 | 4.23 |
| DUSP1 | ENST00000430373 | hsa-miR-6856-5p | 0.97 | 4.23 |
| DUSP1 | ENST00000430373 | hsa-miR-8060 | 0.97 | 4.23 |
| NKAIN1 | ENST00000431759 | hsa-miR-330-5p | 0.97 | 4.21 |
| NKAIN1 | ENST00000431759 | hsa-miR-4640-5p | 0.97 | 4.21 |
| NKAIN1 | ENST00000431759 | hsa-miR-612 | 0.97 | 4.21 |
| TRABD2B | ENST00000553812 | hsa-miR-7851-3p | 0.97 | 4.14 |
| PARP8 | ENST00000456953 | hsa-miR-6852-5p | 0.97 | 4.04 |
| RILPL2 | ENST00000566583 | hsa-miR-6791-5p | 0.97 | 4.01 |
| DNAJA3 | ENST00000671622 | hsa-miR-1301-3p | 0.97 | 3.99 |
| RILPL2 | ENST00000616527 | hsa-miR-589-5p | 0.96 | 3.96 |
| HOMER3 | ENST00000456953 | hsa-miR-4660 | 0.96 | 3.80 |
| HOMER3 | ENST00000456953 | hsa-miR-6752-5p | 0.96 | 3.80 |
| TRAF4 | ENST00000671622 | hsa-miR-106b-5p | 0.96 | 3.79 |
| TRAF4 | ENST00000671622 | hsa-miR-17-5p | 0.96 | 3.79 |
| TRAF4 | ENST00000671622 | hsa-miR-20b-5p | 0.96 | 3.79 |
| FOS | ENST00000430373 | hsa-miR-4747-5p | 0.96 | 3.78 |
| DNAJC22 | ENST00000661332 | hsa-miR-106a-5p | 0.96 | 3.71 |
| DNAJC22 | ENST00000661332 | hsa-miR-106b-5p | 0.96 | 3.71 |
| DNAJC22 | ENST00000661332 | hsa-miR-1224-3p | 0.96 | 3.71 |
| DNAJC22 | ENST00000661332 | hsa-miR-1304-3p | 0.96 | 3.71 |
| DNAJC22 | ENST00000661332 | hsa-miR-143-5p | 0.96 | 3.71 |
| DNAJC22 | ENST00000661332 | hsa-miR-17-5p | 0.96 | 3.71 |
| DNAJC22 | ENST00000661332 | hsa-miR-20a-5p | 0.96 | 3.71 |
| DNAJC22 | ENST00000661332 | hsa-miR-20b-5p | 0.96 | 3.71 |
| DNAJC22 | ENST00000661332 | hsa-miR-373-3p | 0.96 | 3.71 |
| DNAJC22 | ENST00000661332 | hsa-miR-4430 | 0.96 | 3.71 |
| DNAJC22 | ENST00000661332 | hsa-miR-504-3p | 0.96 | 3.71 |
| DNAJC22 | ENST00000661332 | hsa-miR-619-5p | 0.96 | 3.71 |
| DNAJC22 | ENST00000661332 | hsa-miR-93-5p | 0.96 | 3.71 |
| DUSP1 | ENST00000456953 | hsa-miR-6757-5p | 0.96 | 3.69 |
| CRTC1 | ENST00000444346 | hsa-miR-1913 | 0.96 | 3.68 |
| CRTC1 | ENST00000444346 | hsa-miR-296-3p | 0.96 | 3.68 |
| APBB1 | ENST00000553812 | hsa-miR-3192-5p | 0.96 | 3.68 |
| PPIF | ENST00000434411 | hsa-miR-370-5p | 0.96 | 3.66 |
| FHL2 | ENST00000434411 | hsa-miR-18a-3p | 0.95 | 3.62 |
| FHL2 | ENST00000434411 | hsa-miR-6791-5p | 0.95 | 3.62 |
| FHL2 | ENST00000434411 | hsa-miR-7703 | 0.95 | 3.62 |
| PTPN18 | ENST00000434411 | hsa-miR-1233-5p | 0.95 | 3.61 |
| PTPN18 | ENST00000434411 | hsa-miR-3138 | 0.95 | 3.61 |
| PTPN18 | ENST00000434411 | hsa-miR-6720-5p | 0.95 | 3.61 |
| PTPN18 | ENST00000434411 | hsa-miR-6843-3p | 0.95 | 3.61 |
| NPNT | ENST00000553812 | hsa-miR-7851-3p | 0.95 | 3.58 |
| FPGS | ENST00000502301 | hsa-miR-6769b-5p | 0.95 | 3.57 |
| KIFC3 | ENST00000553812 | hsa-miR-6858-5p | 0.95 | 3.55 |
| TPO | ENST00000661332 | hsa-miR-6722-3p | 0.95 | 3.55 |
| TNXB | ENST00000662112 | hsa-miR-4709-3p | 0.95 | 3.54 |
| TNXB | ENST00000662112 | hsa-miR-4747-3p | 0.95 | 3.54 |
| TNXB | ENST00000662112 | hsa-miR-671-5p | 0.95 | 3.54 |
| TNXB | ENST00000662112 | hsa-miR-6799-3p | 0.95 | 3.54 |
| TNXB | ENST00000662112 | hsa-miR-6833-5p | 0.95 | 3.54 |
| TNXB | ENST00000662112 | hsa-miR-6880-5p | 0.95 | 3.54 |
| CRTC1 | ENST00000448058 | hsa-miR-3620-5p | 0.95 | 3.54 |
| SMIM1 | ENST00000662112 | hsa-miR-3192-5p | 0.95 | 3.53 |
| HTT | ENST00000431759 | hsa-miR-330-5p | 0.95 | 3.51 |
| HTT | ENST00000431759 | hsa-miR-4640-5p | 0.95 | 3.51 |
| HTT | ENST00000431759 | hsa-miR-612 | 0.95 | 3.51 |
| PCYT2 | ENST00000431759 | hsa-miR-5088-3p | 0.95 | 3.49 |
| PCYT2 | ENST00000431759 | hsa-miR-6796-5p | 0.95 | 3.49 |
| PCYT2 | ENST00000431759 | hsa-miR-8077 | 0.95 | 3.49 |
| PLBD2 | ENST00000553812 | hsa-miR-30c-2-3p | 0.95 | 3.48 |
| FHL2 | ENST00000566583 | hsa-miR-18a-3p | 0.95 | 3.47 |
| FHL2 | ENST00000566583 | hsa-miR-6791-5p | 0.95 | 3.47 |
| FHL2 | ENST00000566583 | hsa-miR-7703 | 0.95 | 3.47 |
| RAB44 | ENST00000553812 | hsa-miR-30b-3p | 0.95 | 3.46 |
| FPGS | ENST00000671622 | hsa-miR-4524a-3p | 0.95 | 3.42 |
| KIAA2012 | ENST00000661332 | hsa-miR-6827-5p | 0.95 | 3.41 |
| FPGS | ENST00000661332 | hsa-miR-30b-3p | 0.94 | 3.39 |
| FPGS | ENST00000661332 | hsa-miR-6893-5p | 0.94 | 3.39 |
| TNFAIP1 | ENST00000566583 | hsa-miR-7703 | 0.94 | 3.39 |
| ZFP36 | ENST00000434411 | hsa-miR-1972 | 0.94 | 3.38 |
| ZFP36 | ENST00000434411 | hsa-miR-6791-5p | 0.94 | 3.38 |
| ZFP36 | ENST00000434411 | hsa-miR-6808-5p | 0.94 | 3.38 |
| ZFP36 | ENST00000434411 | hsa-miR-939-5p | 0.94 | 3.38 |
| MYCBP2 | ENST00000671622 | hsa-miR-877-3p | 0.94 | 3.37 |
| NSMF | ENST00000431759 | hsa-miR-143-5p | 0.94 | 3.33 |
| NSMF | ENST00000431759 | hsa-miR-4640-5p | 0.94 | 3.33 |
| NSMF | ENST00000431759 | hsa-miR-5589-5p | 0.94 | 3.33 |
| NSMF | ENST00000431759 | hsa-miR-6752-5p | 0.94 | 3.33 |
| TNFAIP1 | ENST00000456953 | hsa-miR-5189-5p | 0.94 | 3.33 |
| TNFAIP1 | ENST00000456953 | hsa-miR-6734-3p | 0.94 | 3.33 |
| PTPN18 | ENST00000566583 | hsa-miR-4738-3p | 0.94 | 3.32 |
| PTPN18 | ENST00000566583 | hsa-miR-6720-5p | 0.94 | 3.32 |
| PTPN18 | ENST00000566583 | hsa-miR-6843-3p | 0.94 | 3.32 |
| RAB44 | ENST00000661332 | hsa-miR-1224-3p | 0.94 | 3.31 |
| RAB44 | ENST00000661332 | hsa-miR-1226-5p | 0.94 | 3.31 |
| RAB44 | ENST00000661332 | hsa-miR-30b-3p | 0.94 | 3.31 |
| RAB44 | ENST00000661332 | hsa-miR-3153 | 0.94 | 3.31 |
| RAB44 | ENST00000661332 | hsa-miR-3689b-3p | 0.94 | 3.31 |
| RAB44 | ENST00000661332 | hsa-miR-3689c | 0.94 | 3.31 |
| RAB44 | ENST00000661332 | hsa-miR-619-5p | 0.94 | 3.31 |
| RAB44 | ENST00000661332 | hsa-miR-6722-3p | 0.94 | 3.31 |
| PDZD4 | ENST00000661332 | hsa-miR-1224-3p | 0.94 | 3.29 |
| PDZD4 | ENST00000661332 | hsa-miR-3619-5p | 0.94 | 3.29 |
| PTPN18 | ENST00000456953 | hsa-miR-1343-3p | 0.94 | 3.29 |
| PTPN18 | ENST00000456953 | hsa-miR-3191-5p | 0.94 | 3.29 |
| CTDSPL | ENST00000662112 | hsa-miR-3192-5p | 0.94 | 3.27 |
| CTDSPL | ENST00000662112 | hsa-miR-4669 | 0.94 | 3.27 |
| CTDSPL | ENST00000662112 | hsa-miR-518c-5p | 0.94 | 3.27 |
| CTDSPL | ENST00000662112 | hsa-miR-6511a-5p | 0.94 | 3.27 |
| CTDSPL | ENST00000662112 | hsa-miR-6829-5p | 0.94 | 3.27 |
| KCNC3 | ENST00000448058 | hsa-miR-3663-3p | 0.94 | 3.26 |
| DNAJC22 | ENST00000671622 | hsa-miR-106b-5p | 0.94 | 3.25 |
| DNAJC22 | ENST00000671622 | hsa-miR-1304-3p | 0.94 | 3.25 |
| DNAJC22 | ENST00000671622 | hsa-miR-17-5p | 0.94 | 3.25 |
| DNAJC22 | ENST00000671622 | hsa-miR-20b-5p | 0.94 | 3.25 |
| DNAJC22 | ENST00000671622 | hsa-miR-212-5p | 0.94 | 3.25 |
| DNAJC22 | ENST00000671622 | hsa-miR-4524a-3p | 0.94 | 3.25 |
| DNAJC22 | ENST00000671622 | hsa-miR-93-5p | 0.94 | 3.25 |
| FAM189B | ENST00000430373 | hsa-miR-4433b-3p | 0.94 | 3.24 |
| FAM189B | ENST00000430373 | hsa-miR-661 | 0.94 | 3.24 |
| ZER1 | ENST00000456953 | hsa-miR-1343-3p | 0.94 | 3.23 |
| ZER1 | ENST00000456953 | hsa-miR-25-5p | 0.94 | 3.23 |
| KIFC3 | ENST00000502301 | hsa-miR-6860 | 0.94 | 3.22 |
| DUSP1 | ENST00000434411 | hsa-miR-3138 | 0.94 | 3.21 |
| SRSF2 | ENST00000566583 | hsa-miR-4738-3p | 0.94 | 3.21 |
| LRRC31 | ENST00000661332 | hsa-miR-6750-5p | 0.94 | 3.19 |
| NPNT | ENST00000431759 | hsa-miR-6851-5p | 0.93 | 3.14 |
| NR5A1 | ENST00000553812 | hsa-miR-3192-5p | 0.93 | 3.12 |
| PTPN18 | ENST00000430373 | hsa-miR-4533 | 0.93 | 3.11 |
| KIFC3 | ENST00000661332 | hsa-miR-1226-5p | 0.93 | 3.11 |
| KIFC3 | ENST00000661332 | hsa-miR-3619-5p | 0.93 | 3.11 |
| NPNT | ENST00000442305 | hsa-miR-4763-3p | 0.93 | 3.11 |
| NPNT | ENST00000442305 | hsa-miR-490-3p | 0.93 | 3.11 |
| NPNT | ENST00000442305 | hsa-miR-7851-3p | 0.93 | 3.11 |
| PXN | ENST00000661332 | hsa-miR-1226-5p | 0.93 | 3.10 |
| PXN | ENST00000661332 | hsa-miR-125a-5p | 0.93 | 3.10 |
| PXN | ENST00000661332 | hsa-miR-3619-5p | 0.93 | 3.10 |
| QSOX1 | ENST00000566583 | hsa-miR-4449 | 0.93 | 3.09 |
| QSOX1 | ENST00000566583 | hsa-miR-6847-5p | 0.93 | 3.09 |
| QSOX1 | ENST00000566583 | hsa-miR-7851-3p | 0.93 | 3.09 |
| KIAA2012 | ENST00000671622 | hsa-miR-212-5p | 0.93 | 3.09 |
| KIAA2012 | ENST00000671622 | hsa-miR-30c-2-3p | 0.93 | 3.09 |
| TRAF4 | ENST00000661332 | hsa-miR-106a-5p | 0.93 | 3.08 |
| TRAF4 | ENST00000661332 | hsa-miR-106b-5p | 0.93 | 3.08 |
| TRAF4 | ENST00000661332 | hsa-miR-17-5p | 0.93 | 3.08 |
| TRAF4 | ENST00000661332 | hsa-miR-20a-5p | 0.93 | 3.08 |
| TRAF4 | ENST00000661332 | hsa-miR-20b-5p | 0.93 | 3.08 |
| TRAF4 | ENST00000661332 | hsa-miR-3619-5p | 0.93 | 3.08 |
| TRAF4 | ENST00000661332 | hsa-miR-4716-3p | 0.93 | 3.08 |
| ZFP36 | ENST00000456953 | hsa-miR-6852-5p | 0.93 | 3.08 |
| PDZD4 | ENST00000431759 | hsa-miR-4640-5p | 0.93 | 3.07 |
| PDZD4 | ENST00000431759 | hsa-miR-5088-3p | 0.93 | 3.07 |
| PDZD4 | ENST00000431759 | hsa-miR-6796-5p | 0.93 | 3.07 |
| SMS | ENST00000430373 | hsa-miR-6765-5p | 0.93 | 3.05 |
| LRRTM1 | ENST00000444346 | hsa-miR-1913 | 0.93 | 3.04 |
| LRRTM1 | ENST00000444346 | hsa-miR-6753-3p | 0.93 | 3.04 |
| TM4SF1 | ENST00000613892 | hsa-miR-3617-3p | 0.92 | 3.00 |
| PPIF | ENST00000456953 | hsa-miR-6769a-5p | 0.92 | 3.00 |
| HTT | ENST00000442305 | hsa-miR-34a-5p | 0.92 | 2.99 |
| HTT | ENST00000442305 | hsa-miR-4763-3p | 0.92 | 2.99 |
| HTT | ENST00000442305 | hsa-miR-6754-5p | 0.92 | 2.99 |
| HTT | ENST00000442305 | hsa-miR-6880-5p | 0.92 | 2.99 |
| VPS37D | ENST00000431759 | hsa-miR-4640-5p | 0.92 | 2.98 |
| VPS37D | ENST00000431759 | hsa-miR-612 | 0.92 | 2.98 |
| NPNT | ENST00000661332 | hsa-miR-6722-3p | 0.92 | 2.98 |
| TIGD5 | ENST00000661332 | hsa-miR-1224-3p | 0.92 | 2.97 |
| TIGD5 | ENST00000661332 | hsa-miR-6722-3p | 0.92 | 2.97 |
| LRCOL1 | ENST00000671622 | hsa-miR-4524a-3p | 0.92 | 2.96 |
| TRABD2B | ENST00000444346 | hsa-miR-296-3p | 0.92 | 2.95 |
| RAB44 | ENST00000671622 | hsa-miR-212-5p | 0.92 | 2.92 |
| PPIF | ENST00000566583 | hsa-miR-370-5p | 0.92 | 2.92 |
| TRABD2B | ENST00000671622 | hsa-miR-1301-3p | 0.92 | 2.91 |
| TRABD2B | ENST00000671622 | hsa-miR-17-5p | 0.92 | 2.91 |
| TRABD2B | ENST00000671622 | hsa-miR-20b-5p | 0.92 | 2.91 |
| TRABD2B | ENST00000671622 | hsa-miR-6758-5p | 0.92 | 2.91 |
| TRABD2B | ENST00000671622 | hsa-miR-93-5p | 0.92 | 2.91 |
| TNXB | ENST00000456953 | hsa-miR-1266-5p | 0.92 | 2.91 |
| TNXB | ENST00000456953 | hsa-miR-1343-3p | 0.92 | 2.91 |
| TNXB | ENST00000456953 | hsa-miR-3184-3p | 0.92 | 2.91 |
| TNXB | ENST00000456953 | hsa-miR-5189-5p | 0.92 | 2.91 |
| TNXB | ENST00000456953 | hsa-miR-671-5p | 0.92 | 2.91 |
| TNXB | ENST00000456953 | hsa-miR-6752-5p | 0.92 | 2.91 |
| TNXB | ENST00000456953 | hsa-miR-6862-3p | 0.92 | 2.91 |
| RILPL2 | ENST00000430373 | hsa-miR-6871-5p | 0.92 | 2.90 |
| FANCC | ENST00000661332 | hsa-miR-17-5p | 0.92 | 2.90 |
| FANCC | ENST00000661332 | hsa-miR-20a-5p | 0.92 | 2.90 |
| FANCC | ENST00000661332 | hsa-miR-20b-5p | 0.92 | 2.90 |
| FANCC | ENST00000661332 | hsa-miR-3153 | 0.92 | 2.90 |
| FANCC | ENST00000661332 | hsa-miR-3619-5p | 0.92 | 2.90 |
| FANCC | ENST00000661332 | hsa-miR-504-3p | 0.92 | 2.90 |
| FANCC | ENST00000661332 | hsa-miR-619-5p | 0.92 | 2.90 |
| FANCC | ENST00000661332 | hsa-miR-6893-5p | 0.92 | 2.90 |
| FANCC | ENST00000661332 | hsa-miR-93-5p | 0.92 | 2.90 |
| PLBD2 | ENST00000661332 | hsa-miR-6811-5p | 0.92 | 2.88 |
| PLBD2 | ENST00000661332 | hsa-miR-6814-5p | 0.92 | 2.88 |
| PLBD2 | ENST00000661332 | hsa-miR-6827-5p | 0.92 | 2.88 |
| PLBD2 | ENST00000661332 | hsa-miR-6870-5p | 0.92 | 2.88 |
| PLB1 | ENST00000444346 | hsa-miR-4690-3p | 0.92 | 2.88 |
| ZFP36 | ENST00000566583 | hsa-miR-6791-5p | 0.92 | 2.88 |
| ZNF574 | ENST00000661332 | hsa-miR-6722-3p | 0.92 | 2.87 |
| ZNF574 | ENST00000661332 | hsa-miR-93-5p | 0.92 | 2.87 |
| KLC2 | ENST00000431759 | hsa-miR-4640-5p | 0.92 | 2.86 |
| KLC2 | ENST00000431759 | hsa-miR-5589-5p | 0.92 | 2.86 |
| KLC2 | ENST00000431759 | hsa-miR-7109-5p | 0.92 | 2.86 |
| UBALD1 | ENST00000553812 | hsa-miR-3192-5p | 0.91 | 2.83 |
| ZER1 | ENST00000434411 | hsa-miR-1233-5p | 0.91 | 2.83 |
| ZER1 | ENST00000434411 | hsa-miR-6791-5p | 0.91 | 2.83 |
| ZER1 | ENST00000434411 | hsa-miR-6808-5p | 0.91 | 2.83 |
| ZER1 | ENST00000434411 | hsa-miR-6843-3p | 0.91 | 2.83 |
| ZER1 | ENST00000434411 | hsa-miR-922 | 0.91 | 2.83 |
| TNXB | ENST00000566583 | hsa-miR-18a-3p | 0.91 | 2.83 |
| TNXB | ENST00000566583 | hsa-miR-4449 | 0.91 | 2.83 |
| TNXB | ENST00000566583 | hsa-miR-6720-5p | 0.91 | 2.83 |
| TNXB | ENST00000566583 | hsa-miR-922 | 0.91 | 2.83 |
| PCYT2 | ENST00000442305 | hsa-miR-6754-5p | 0.91 | 2.82 |
| EMD | ENST00000430373 | hsa-miR-8060 | 0.91 | 2.82 |
| PARP8 | ENST00000430373 | hsa-miR-299-3p | 0.91 | 2.81 |
| TIGD5 | ENST00000442305 | hsa-miR-4763-3p | 0.91 | 2.81 |
| TIGD5 | ENST00000442305 | hsa-miR-5787 | 0.91 | 2.81 |
| MYCBP2 | ENST00000661332 | hsa-miR-125a-5p | 0.91 | 2.81 |
| MYCBP2 | ENST00000661332 | hsa-miR-345-5p | 0.91 | 2.81 |
| MYCBP2 | ENST00000661332 | hsa-miR-4716-3p | 0.91 | 2.81 |
| MYCBP2 | ENST00000661332 | hsa-miR-619-5p | 0.91 | 2.81 |
| MYCBP2 | ENST00000661332 | hsa-miR-6811-5p | 0.91 | 2.81 |
| MYCBP2 | ENST00000661332 | hsa-miR-6893-5p | 0.91 | 2.81 |
| SLC10A3 | ENST00000434411 | hsa-miR-6780a-5p | 0.91 | 2.79 |
| NSMF | ENST00000442305 | hsa-miR-4763-3p | 0.91 | 2.79 |
| NSMF | ENST00000442305 | hsa-miR-5787 | 0.91 | 2.79 |
| PAX7 | ENST00000431759 | hsa-miR-6752-5p | 0.91 | 2.79 |
| TNFAIP1 | ENST00000430373 | hsa-miR-939-5p | 0.91 | 2.77 |
| FPGS | ENST00000553812 | hsa-miR-30b-3p | 0.91 | 2.77 |
| AK1 | ENST00000566583 | hsa-miR-30c-2-3p | 0.91 | 2.76 |
| AK1 | ENST00000566583 | hsa-miR-6720-5p | 0.91 | 2.76 |
| AK1 | ENST00000566583 | hsa-miR-6843-3p | 0.91 | 2.76 |
| AK1 | ENST00000566583 | hsa-miR-7851-3p | 0.91 | 2.76 |
| APBB1 | ENST00000661332 | hsa-miR-4716-3p | 0.91 | 2.75 |
| APBB1 | ENST00000661332 | hsa-miR-6827-5p | 0.91 | 2.75 |
| APBB1 | ENST00000661332 | hsa-miR-6893-5p | 0.91 | 2.75 |
| DUS2 | ENST00000661332 | hsa-miR-4457 | 0.91 | 2.75 |
| DUS2 | ENST00000661332 | hsa-miR-4756-5p | 0.91 | 2.75 |
| DUS2 | ENST00000661332 | hsa-miR-6754-5p | 0.91 | 2.75 |
| TNFAIP1 | ENST00000434411 | hsa-miR-6808-5p | 0.91 | 2.75 |
| TNFAIP1 | ENST00000434411 | hsa-miR-7703 | 0.91 | 2.75 |
| TNFAIP1 | ENST00000434411 | hsa-miR-939-5p | 0.91 | 2.75 |
| ABCC10 | ENST00000671622 | hsa-miR-4524a-3p | 0.91 | 2.73 |
| ABCC10 | ENST00000671622 | hsa-miR-6758-5p | 0.91 | 2.73 |
| ABCC10 | ENST00000671622 | hsa-miR-8060 | 0.91 | 2.73 |
| NKAIN1 | ENST00000502301 | hsa-miR-6860 | 0.91 | 2.73 |
| TRABD2B | ENST00000502301 | hsa-miR-6860 | 0.91 | 2.71 |
| FAM189B | ENST00000456953 | hsa-miR-6752-5p | 0.91 | 2.71 |
| FOS | ENST00000456953 | hsa-miR-1266-5p | 0.90 | 2.69 |
| FOS | ENST00000456953 | hsa-miR-3130-3p | 0.90 | 2.69 |
| FOS | ENST00000456953 | hsa-miR-4749-3p | 0.90 | 2.69 |
| FOS | ENST00000456953 | hsa-miR-6734-3p | 0.90 | 2.69 |
| SLC10A3 | ENST00000456953 | hsa-miR-1266-5p | 0.90 | 2.69 |
| SLC10A3 | ENST00000456953 | hsa-miR-1343-3p | 0.90 | 2.69 |
| ZER1 | ENST00000566583 | hsa-miR-6791-5p | 0.90 | 2.67 |
| ZER1 | ENST00000566583 | hsa-miR-6843-3p | 0.90 | 2.67 |
| ZER1 | ENST00000566583 | hsa-miR-922 | 0.90 | 2.67 |
| FAM189B | ENST00000613892 | hsa-miR-6826-3p | 0.90 | 2.66 |
| PCYT2 | ENST00000553812 | hsa-miR-30b-3p | 0.90 | 2.66 |
| TRABD2B | ENST00000448058 | hsa-miR-3620-5p | 0.90 | 2.65 |
| TIGD5 | ENST00000671622 | hsa-miR-8060 | 0.90 | 2.64 |
